# Structural basis for chitin acquisition by marine *Vibrio* species

**DOI:** 10.1038/s41467-017-02523-y

**Published:** 2018-01-15

**Authors:** Anuwat Aunkham, Michael Zahn, Anusha Kesireddy, Karunakar Reddy Pothula, Albert Schulte, Arnaud Baslé, Ulrich Kleinekathöfer, Wipa Suginta, Bert van den Berg

**Affiliations:** 1 Biochemistry-Electrochemistry Research Unit, Institute of Science, Suranaree University of Technology, Nakhon Ratchasima, 30000 Thailand; 2 Institute for Cell and Molecular Biosciences, The Medical School, Newcastle University, Newcastle upon Tyne, NE2 4HH UK; 3 Department of Physics & Earth Sciences, Jacobs University Bremen, 28759 Bremen, Germany; 4 School of Biomolecular Science and Engineering, Vidyasirimedhi Institute of Science and Technology (VISTEC), Wangchan Valley, 555 Moo 1 Payupnai, Wangchan, Rayong 21210 Thailand; 5 Center of Excellence in Advanced Functional Materials, Suranaree University of Technology, Nakhon Ratchasima, 30000 Thailand

## Abstract

Chitin, an insoluble polymer of *N*-acetylglucosamine, is one of the most abundant biopolymers on Earth. By degrading chitin, chitinolytic bacteria such as *Vibrio harveyi* are critical for chitin recycling and maintenance of carbon and nitrogen cycles in the world’s oceans. A decisive step in chitin degradation is the uptake of chito-oligosaccharides by an outer membrane protein channel named chitoporin (ChiP). Here, we report X-ray crystal structures of ChiP from *V. harveyi* in the presence and absence of chito-oligosaccharides. Structures without bound sugar reveal a trimeric assembly with an unprecedented closing of the transport pore by the N-terminus of a neighboring subunit. Substrate binding ejects the pore plug to open the transport channel. Together with molecular dynamics simulations, electrophysiology and in vitro transport assays our data provide an explanation for the exceptional affinity of ChiP for chito-oligosaccharides and point to an important role of the N-terminal gate in substrate transport.

## Abstract (web summary)

Chitin degrading bacteria are important for marine ecosystems. Here the authors structurally and functionally characterize the *Vibrio harveyi* outer membrane diffusion channel chitoporin and give mechanistic insights into chito-oligosaccharide uptake.

## Introduction

Chitin, an insoluble polymer of β1,4-linked *N*-acetylglucosamine (GlcNAc) residues, is one of the most abundant biopolymers on Earth, along with cellulose^1,2^. Chitin serves as the major structural component of arthropods, the cell walls of some fungi, and the primary exoskeletons of crustaceans and marine zooplankton^3^. The global estimation of chitin production is ~10^10^–10^11^ tons^4^. Remarkably, while providing a constant rain of polysaccharides to the ocean floor (“marine snow”), no substantial accumulation of chitin in ocean sediments occurs due to the rapid recycling of chitin driven by chitinolytic bacteria, mainly from the family Vibrionaceae^5,6^. These bacteria utilize only chitin as a sole source of cellular energy, and certain *Vibrio* species such as *Vibrio harveyi* and *Vibrio parahaemolyticus* are extremely fast growing. Hence, these bacteria play critical roles in maintaining the carbon and nitrogen cycles in marine ecosystems.

The chitin utilization pathway of marine *Vibrios* is highly conserved and incorporates a number of characterized and uncharacterized enzymes, chitin binding proteins, and transport proteins^7,8^. Proteins involved in the initial processes include chemotaxis proteins responsive to chitin oligosaccharides and extracellular chitinases that degrade the insoluble polymer into water-soluble chitooligosaccharides that are imported across the outer membrane (OM) through a dedicated, chitooligosaccharide-specific channel^9–11^. In the periplasmic space, chitin dextrinase^12^ and β-*N*-acetylglucosaminidase^13,14^ generate the mono- (GlcNAc) and disaccharide (GlcNAc_2_) that are subsequently transported into the cytoplasm via several ABC transporter complexes in the inner membrane. Finally, at least six cytoplasmic enzymes convert the transport products to fructose-6-P, NH_3_, and acetate^7^.

Cloning of a number of chitin utilization genes has been reported from *Vibrio furnissii*, *Vibrio cholera*, and *V. harveyi* and the corresponding proteins have been characterized^9,10,12,15–20^. A key step in chitin utilization represents the cellular acquisition of soluble chitin oligosaccharides (GlcNAc_2–6_) produced by the action of extracellular chitinases^20^. This uptake process is carried out by an OM diffusion channel, termed chitoporin (ChiP)^9–11^. ChiP was first identified in *V. furnissii* and was proposed to be a chitooligosaccharide-uptake channel based on expression profiles in the presence of various sugar substrates^21^. DNA microarray expression profiles further demonstrated that expression of the *chiP* gene in *V. cholerae* is induced by chitin oligosaccharides and that the genes responsible for chitin degradation are under the stringent control of the *chiS* regulon^21^. The gene encoding for ChiP is found in most marine members of the Vibrionaceae, emphasizing the general importance of this OM diffusion channel for chitin utilization.

On a molecular level, it is so far unclear how ChiP mediates the specific uptake of chitooligosaccharides. The protein from *V. harveyi* (denoted *Vh*ChiP) has recently been cloned and expressed at high levels in *Escherichia coli* and purified to homogeneity. *Vh*ChiP was purified as a stable trimer, suggesting a structural similarity to the general diffusion porins OmpF and OmpC from *E. coli*. In single-channel electrophysiology, *Vh*ChiP displays a trimeric conductance of 1.8 nS in 1 M KCl, which is half of the trimeric conductance of *E. coli* OmpF (4.2 nS)^22^ but substantially larger than its well-characterized sugar-specific analog LamB (maltoporin) from *E. coli* (160 pS) and *Salmonella typhimurium* (90 pS)^23,24^. The addition of micromolar concentrations of chitooligosaccharides such as chitohexaose (GlcNAc_6_) causes a complete block of ionic current, demonstrating tight binding. Interestingly, closely related sugars such as maltohexaose do not interact with the channel, suggesting remarkable substrate specificity of *Vh*ChiP. The binding constant (*K*) for the most potent substrate chitohexaose is about 5 × 10^6^ M^−1^, which is at least an order of magnitude higher compared to other OM sugar-specific diffusion channels^11^. In vitro liposome swelling assays confirm the highly specific nature of the channel for chitooligosaccharides^10^. Statistical analysis on stochastic fluctuations of ion current through *Vh*ChiP in the presence of chitohexaose indicated that *Vh*ChiP has multiple binding sites for sugar, and the trapping properties of chitoporin exhibit memory effects, given that the average binding rate of an unblocked monomer is larger when its neighboring monomers are blocked. Such results suggested a possible design strategy to enhance the rate of sugar uptake by the bacterium^25,26^. A homology model based on the X-ray crystal structure of *Delftia acidovorans* Omp32 (pdb ID: 2FGR)^27^ predicted Trp136, located at the mouth of the channel constriction, to be an important residue for chitin transport^28^. This notion was confirmed by single-channel electrophysiology and liposome swelling experiments, showing that for the preferred substrate chitohexaose, mutation of Trp136 led to decreased binding affinity and reduced rates of uptake respectively.

Here, we report X-ray crystal structures of *Vh*ChiP in the absence and presence of chitooligosaccharides. Together with single-channel electrophysiology and molecular dynamics (MD) simulations the results reveal the structural basis for the exquisite substrate specificity of chitoporin and clarify the mechanism of facilitated diffusion of chitooligosaccharides across the OM of Vibrionaceae.

## Results

### Purification and crystallization of *Vh*ChiP

**Fig. 1:**
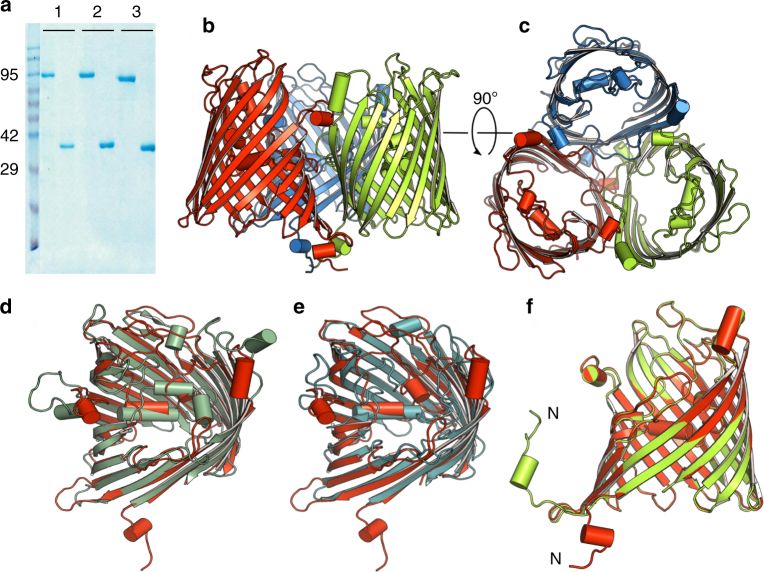
X-ray crystal structure of in vitro-folded *Vh*ChiP. **a** SDS-PAGE gel of OM-expressed (native; 1), in vitro-folded (2) and truncated *Vh*ChiP (3) loaded both as non-boiled (left lanes) and boiled samples (right lanes). **b** Side view and **c** top view cartoon presentations of the in vitro-folded *Vh*ChiP trimer. **d**, **e** Top view superpositions of in vitro-folded *Vh*ChiP (red) with *Neisseria meningitidis* PorB (green; **d**) and *Delftia acidovorans* Omp32 (blue; **e**). **f** Superposition of in vitro-folded *Vh*ChiP (red) with OM-expressed *Vh*ChiP (green). The N-termini have been labeled

We expressed *Vh*ChiP without a His-tag in the OM of the porin-deficient *E. coli* Bl21 omp8 strain and obtained reasonable yields (~3 mg from 12 l of culture) even though *Vh*ChiP overexpression appeared to be highly toxic for *E. coli*. The protein was expressed without a tag based on the predicted similarity to general porins in which the N- and C-termini of the monomers interact to form a salt bridge. Figure 1a shows that boiled and non-boiled SDS-PAGE samples have different mobilities, with trimers observed in non-boiled samples and monomers after boiling, i.e., *Vh*ChiP is heat-modifiable. The purified protein was crystallized in the presence of 0.4% C_8_E_4_ as detergent (Methods). The obtained crystals of *Vh*ChiP in space group C2 diffracted to reasonable resolutions of ~2.5 Å (Supplementary Table 1). Despite this, structure solution using molecular replacement (MR) failed, presumably due to the lack of a good homology model given that sequence identities of likely search models with *Vh*ChiP are 20% or less. Soaking of native crystals with different heavy atoms was also not successful. Next, we attempted to express *Vh*ChiP in minimal media in the presence of seleno-methionine (SeMet) for phasing using single/multiple anomalous dispersion (SAD/MAD) approaches, but the cells failed to grow in accordance with the protein toxicity that was observed in rich medium. Subsequently we cloned *Vh*ChiP without a signal sequence and with a C-terminal His_6_-tag into pET28a (Methods) to express the protein into inclusion bodies (IBs) and avoid toxicity issues. As expected, the cells grew now very well in minimum medium in the presence of SeMet. *Vh*ChiP from IBs was folded in vitro from 8 M urea and purified as the native protein (Methods). Well-diffracting crystals (~ 2 Å resolution) in space group P2_1_ were obtained and a SAD data set was collected at the absorption edge of Selenium. The structure was solved using Autosol in Phenix and refined in Refmac (Methods).

### *Vh*ChiP forms trimers with N-terminally plugged pores

In vitro-folded *Vh*ChiP forms a trimeric assembly of oval β-barrels, each consisting of 16 strands (Fig. 1), like the general diffusion porins OmpF/C from *E. coli*. Unlike OmpF/C however, the N-terminus of *Vh*ChiP does not form an intramolecular salt bridge with the C-terminal carboxyl group. Instead, the first N-terminal 17 amino acids of *Vh*ChiP are not part of the barrel and extend into the periplasm where they form a trimerization motif like that observed in the phosphate transporter OprP from *Pseudomonas aeruginosa*^29^. The first nine amino acids of the N-terminus are disordered and not visible in the electron density (Fig. 1b). The overall topology of the barrel, including the periplasmic N-terminal region, is correctly predicted by Boctopus and PRED-TMBB^30,31^. A DALI search^32^ identifies *N. meningitidis* PorB, which has 18% sequence identity to *Vh*ChiP, as the closest structural homolog in the database (*Z* = 29; r.m.s.d. 2.7 Å over 301 residues). The second-highest similarity is observed for Omp32 from *D. acidovorans* (*Z* = 26; r.m.s.d. 2.7 Å over 295 residues), which was used for previous homology modeling of *Vh*ChiP. Overall, the differences between both structural homologs and *Vh*ChiP are substantial, especially for most extracellular loops and the functionally important, barrel constricting loop L3 (Fig. 1 d, e).

**Fig. 2:**
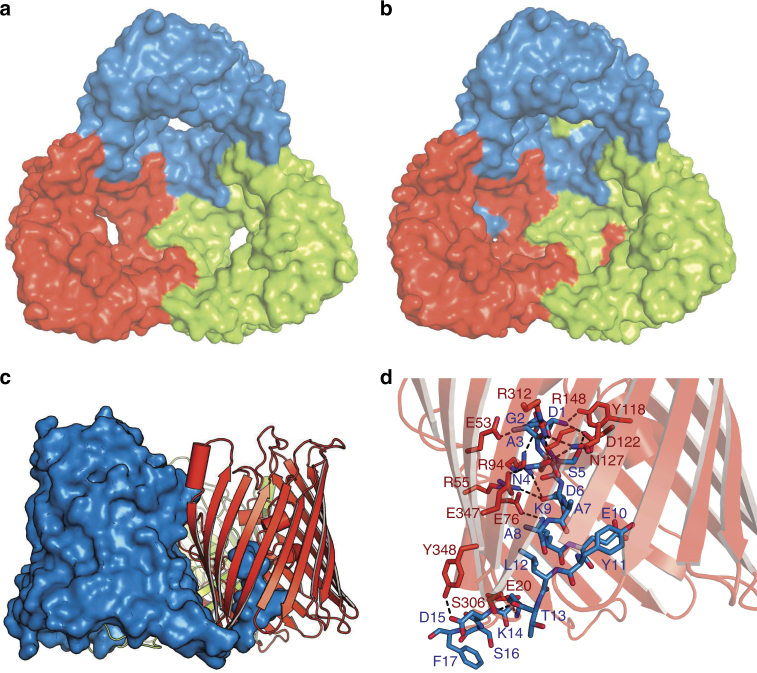
OM-expressed *Vh*Chip has N-terminally plugged channels. **a**, **b** Surface representations (top view) of in vitro-folded *Vh*ChiP (**a**) and natively expressed *Vh*ChiP (**b**). The barrel lumen of the natively expressed *Vh*ChiP is occupied by the N-terminus of a neighbor barrel. **c** N-terminal insertion mode of natively expressed *Vh*ChiP. The N-terminus of one barrel (blue) plugs the lumen of a neighbor barrel (red). **d** Polar interactions between the inner barrel wall (red) with the N-terminus of a neighbor barrel (blue)

The crystal structure of the OM-expressed channel is almost identical to that of the in vitro-folded protein. Strikingly however, in OM-expressed *Vh*ChiP the N-termini plug the pore of a neighboring β-barrel within the trimeric assembly, effectively blocking all β-barrels for substrate transport (Fig. 2). The blocking of a channel by a neighbor within an oligomeric assembly is, to our knowledge, unprecedented. The reason that the plug is inter- and not intra-molecular might be due to fact that the N-terminal β-strand is pointing away from its own pore towards the neighboring one (Fig. 1f and Supplementary Fig. 1). This is due to the small angle of the strand(s) relative to the OM plane. Thus, to plug its own pore, the N-terminus would have to fold back on itself in a rather elaborate way and would likely need to be longer. All residues of the N-terminus have good electron density in the structure (Supplementary Fig. 1) and a number of polar interactions can be identified between the N-terminal plug and the barrel, most notably between Asp1 (carboxylate)-Arg94/148, Asp1 (α-amino group)-Y118/Asp122, Ala3-Glu53, Asn4-Asn127/Arg312/Glu347, Ser5-Asp122, Asp6-Arg94, and Lys9-Glu76 (Fig. 2). Thus, five out of the first six residues of the N-terminus make polar interactions with the pore of the protein, suggesting that the plug is stably inserted in the pore. It should be noted that for in vitro-folded *Vh*ChiP, two residues (MG) were added to the N-terminus of the mature protein for inclusion body expression, and it is possible that the presence of those additional residues has destabilized the plug sufficiently for it be outside the pore.

**Fig. 3:**
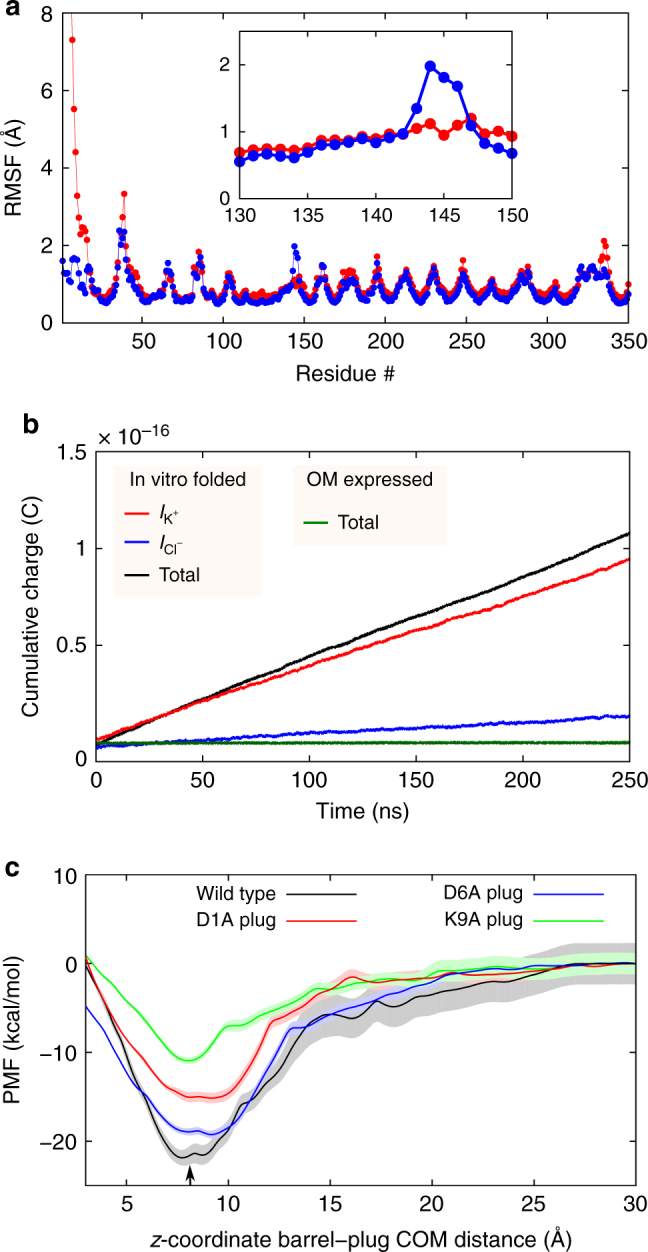
Behavior of the pore plug in MD simulations. **a** Average Cα RMSF of the protein dynamics in vitro-folded (red) and OM-expressed (blue) *Vh*ChiP. In addition to the differences for the first 14 residues, a difference in the RMSF for residues 143–146 in loop L3 can be clearly observed. **b** Cumulative total charge as a function of time for a 1 M KCl solution at 200 mV. In addition, the cumulative charges for K^+^ and Cl^−^ ions separately are shown as well. The data was averaged over the three simulations and the slopes of these curves yield the respective currents. **c** Free-energy surfaces of unbinding of the N-terminus in wild type and mutant channels. The shaded areas indicate the respective error estimates. The arrow represents the position of the N-terminal plug in the crystal structure of OM-expressed *Vh*ChiP

To explore the stability of the pore plug further, we performed 500 ns equilibrium MD simulations of in vitro-folded and natively expressed *Vh*ChiP, with free periplasmic and pore-inserted N-termini respectively. The N-terminal ten residues of in vitro-folded *Vh*ChiP are highly flexible and do not insert into the pore (Fig. 3). As expected from the interactions observed in the crystal structure, the plug in natively expressed *Vh*ChiP is very stable with root mean square fluctuations (RMSF) of about 1.5 Å. Interestingly, in the presence of the pore plug, residues 143–146 located in loop L3 show enlarged RMSF values (Fig. 3a). The movements in loop L3 are likely to be induced by fluctuations of the tail of the plug within the pore, and we hypothesize that these concerted motions might initiate the N-terminal unbinding process that has to occur in order for substrate to bind.

In a next step, MD simulations (3 × 250 ns) were performed to estimate the conductance of the in vitro-folded channel in a 1 M KCl solution in the presence of a 200 mV membrane potential. Consistent with the experiments, the in vitro-folded trimeric channel shows a theoretical conductance value of 2.13 ± 0.15 nS, only slightly higher than the experimental value of 1.8 nS measured at lower voltages^22^. When the plug is inside the pore, only very few ions can pass the plug and no statistically meaningful current value can be determined in the simulation time (Fig. 3b).

To obtain a more quantitative result concerning the binding energies of the N-termini, we determined one-dimensional free-energy surfaces for wild type and D1A, D6A and K9A N-terminal plug mutants (Methods) using the umbrella sampling technique with two 2.24 µs long simulations. The mutated residues are involved in electrostatic interactions with channel-lining residues (Figs 2 and 3) and are therefore likely to be important for the stability of the plug-inserted state. As can be seen in Fig. 3c, the binding energy of the wild type N-terminus is about 22 kcal mol^−1^ and the profile shares similar features as that obtained recently for the monomeric cyclodextrin channel CymA^33^. The D6A and D1A mutants had relatively little effect, while the K9A mutant reduced the binding energy roughly by a factor of two (Fig. 3c). The result suggests that Lys9 plays an important role in the stability of the plug-inserted state. Intriguingly, in CymA it has been verified experimentally that a similar positive residue is involved in the stability of the plug-bound conformation^33^. It is important to note that all determined free-energy surfaces show only one minimum, which is for the N-terminus located inside the pore. This strongly supports the stability of the closed state of the *Vh*ChiP channel as observed in the crystal structure of the natively expressed protein.

### Chitohexaose binding to in vitro-folded *Vh*ChiP

**Fig. 4:**
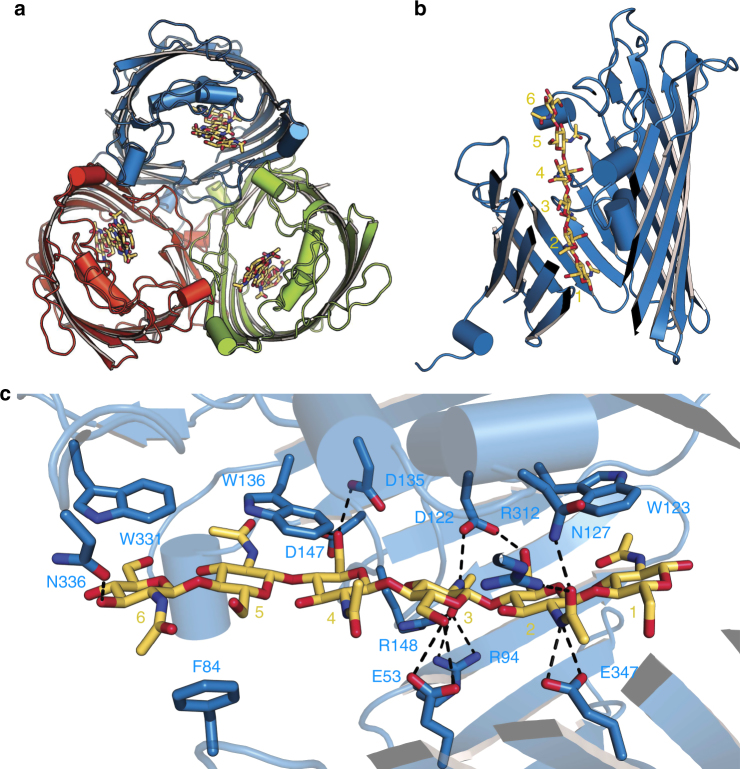
Chitohexaose binding to in vitro-folded *Vh*ChiP. **a** Overview from the extracellular side. **b** Slabbed view from the side, with substrate residues labeled. **c** Schematic, showing interactions of key *Vh*ChiP residues with chitohexaose

To obtain a structure of *Vh*ChiP in complex with a substrate, we co-crystallized in vitro-folded *Vh*ChiP with the substrate chitohexaose. Well-diffracting crystals grew under the same conditions and in the same space group as for the apo protein (Supplementary Table 1). Following MR, density was observed for all six units of the GlcNAc_6_ oligosaccharide (Fig. 4 and Supplementary Fig. 2). The substrate is bound in an extended conformation, with the density for the GlcNac units at both ends of the substrate (GlcNac-1 and -6) somewhat less clear compared to that for the central GlcNac-2–GlcNac-5 units. However, the density is of sufficient quality for placement of the substrate molecule with its reducing end (denoted GlcNAc-1) on the periplasmic side (Supplementary Fig. 2), stacked against the aromatic ring of Trp123. On the extracellular side of the channel constriction, the last sugar unit (denoted GlcNAc-6) stacks against Trp331 (Fig. 4). The same orientation of substrate (reducing end periplasmic) has been observed for maltoporin, albeit at lower resolution (2.8 Å)^34^.

**Fig. 5:**
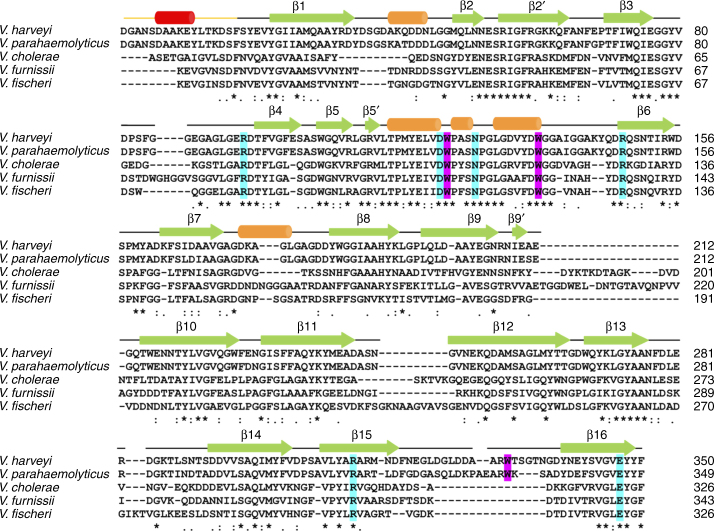
Alignment of *Vh*ChiP orthologs from *Vibrio* species. Observed secondary structure elements have been indicated (orange cylinders, helices; green arrows, β-sheets). The first helix that is part of the N-terminal plug is presented in red. Key aromatic (purple) and hydrophilic residues (cyan) interacting with chitooligosaccharides are colored. The following orthologs have been aligned: *V. harveii* (GenBank ID: HF558985.1), *V. parahaemolyticus* (GenBank ID: CPO12950.1), *V. cholera* (GenBank ID: DQ774012.1), *V. furnissii* (GenBank ID: AF129934.1), and *V. fischeri* (GenBank ID: CP001139.1). Chitoporin from *V. harveyi* (UniProtKB/TrEMBL entry: L0RVU0) was used as protein template to identify putative chitoporins. The alignment was generated using “CLUSTALW” algorithm in the DNASTAR package and displayed in Genedoc. The secondary structure of *Vh*ChiP was generated by ESPript v. 2.2

Besides the ring stacking, few other interactions are present between GlcNAc-1 and GlcNAc-6 and *Vh*ChiP. The aromatic ring of Trp136 stacks against both GlcNAc-4 and GlcNAc-5, providing a clear explanation as to why this residue is important for chitohexaose binding by *Vh*ChiP^28^. Both Trp123 and Trp136 are conserved in *Vh*ChiP orthologs, whereas Trp331 is not (Fig. 5). The structure provides a clear rationale for the fact that (GlcNAc_6_) is the best substrate for *Vh*ChiP, since beyond GlcNAc-1 and GlcNAc-6 the channel widens, decreasing the potential for interactions between the substrate and the channel. The central four residues of the sugar chain (GlcNAc-2 to GlcNAc-5) form several polar interactions with the channel interior, some of which are mediated by water molecules (Fig. 4). Importantly, the density for the central part of GlcNAc_6_ is well-defined and allows unambiguous assignment of the acetamido groups, which for successive GlcNAc units point in opposite directions, as also observed for the chitohexaose molecule bound to the *E. coli* transglycosidase MltA^35^. The acetamido carbonyl groups of GlcNAc-2 and GlcNAc-3 are likely to be especially important for binding. The former interacts closely with the amide of Asn127 and to a lesser extent with Arg312, whereas the latter makes strong hydrogen bonds to the side chains of Arg94 and Arg148 (Fig. 4). In addition, the acetamido amides of GlcNAc-2 and GlcNAc-3 interact with the carboxylates of Glu347 and Asp122 respectively.

### Chitotetraose binding to natively expressed *Vh*ChiP

Given that the channels of the OM-expressed apo-protein are closed due to the insertion of the N-terminus of a neighboring monomer (Fig. 2), it is important to establish whether the incoming substrate from the extracellular space could displace the N-terminus to bind in the pore. In addition, we wanted to establish how chitooligosaccharides of different lengths bind to *Vh*ChiP. To answer these questions we co-crystallized OM-expressed *Vh*ChiP with chitotetraose (GlcNAc_4_). OM-expressed *Vh*ChiP in the presence of the GlcNAc_4_ substrate also crystallizes under the same conditions and in the same space group as the closed apo-protein (Supplementary Table 1). Importantly, the oligosaccharide is bound in the channel and has displaced the N-terminus, which is now in the periplasmic space and has the same conformation as that of the in vitro-folded protein.

**Fig. 6:**
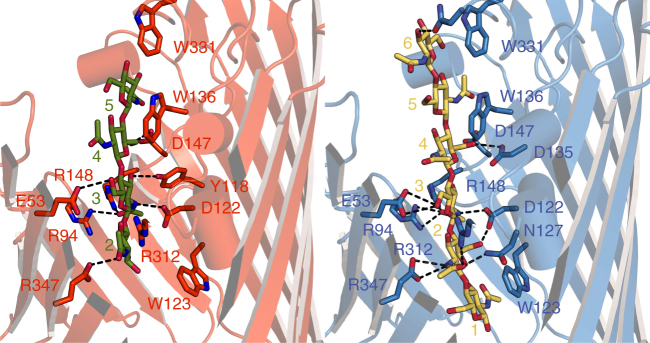
Chitotetraose binding to *Vh*ChiP displaces the N-terminal plug. Side views in the same orientations, showing the bound chitotetraose in natively expressed *Vh*ChiP (left panel) and chitohexaose in in vitro-folded *Vh*ChiP (right panel). Residues interacting with the sugars are labeled

The GlcNAc_4_ substrate occupies approximately the positions of GlcNAc-2 to GlcNAc-5 of the GlcNAc_6_ chain (Fig. 6), suggesting that the central positions of the binding site provide the most binding energy for chitooligosaccharides (note that the subunits of GlcNAc_4_ are numbered 2–5 to provide consistency with those of chitohexaose). However, there are differences of up to 5 Å for individual atoms between the two structures, especially at both ends of the substrate (GlcNAc-2 and GlcNAc-5). In most cases, however, this does not dramatically change the interactions between the sugar and the channel. For example, the acetamido carbonyl group GlcNAc-2 interacts in both structures with Asn127 and Arg312 whereas that of GlcNAc-3 interacts with Arg94/148 (Fig. 6). It appears therefore that *Vh*ChiP accommodates compounds of different length via subtle changes in the way the substrates are bound. For compounds of four GlcNAc units or larger, the central binding sites (2–5) are likely to be always occupied, in a manner as observed in our structures. However, since X-ray crystallography is an averaging technique it is possible that the binding register may vary, i.e., small populations of, e.g., chitohexaose might be bound with GlcNAc-1 at position 2 (with GlcNAc-6 being disordered). As alluded to above, it is also possible that mixed populations are present with regards to the orientation of the substrate, i.e., the reducing end might be extracellular in part of the population. Future MD simulations could shed light on these details.

### Ejection of the pore plug in electrophysiology

**Fig. 7:**
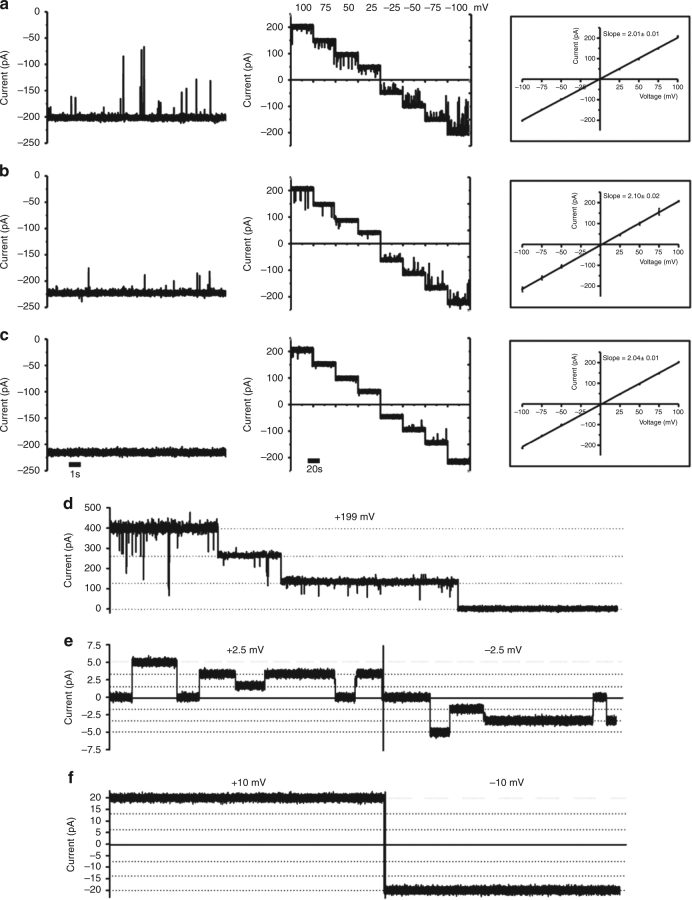
Voltage-induced ejection of the pore plug in *Vh*ChiP. **a**–**c** Single-channel electrophysiology of OM-folded *Vh*ChiP (**a**), in vitro-folded *Vh*ChiP (**b**) and truncated *Vh*ChiP (**c**). Left panels show typical traces at −100 mV, whereas the center and right panels show the current–voltage profiles. Data represent mean ± s.d., *n* = 3. **d** Stepwise voltage-induced closure of OM-folded *Vh*ChiP channels at 200 mV. **e**, **f** Typical traces observed at ±2.5 mV (**e**) and at ±10 mV (**f**). The data shown in **d**–**f** are for the same experiment, with protein only added at the start (**d)**. Traces shown are representative of three independent experiments

To probe the role of the N-terminus in substrate binding and transport in more detail we carried out single-channel electrophysiological experiments for in vitro-folded and OM-expressed *Vh*ChiP. Based on the crystal structure we also generated a *Vh*ChiP variant with the first 19 N-terminal residues removed, denoted truncated *Vh*ChiP. At standard electrophysiological conditions with applied potentials of −100 and +100 mV, the single-channel conductance is essentially the same (~2.0 nS) for all three *Vh*ChiP proteins (Fig. 7a–c), and are fully consistent with data for the native protein obtained in previous studies^10,11^. Surprisingly, there is no clear evidence of a closed channel for any of the three *Vh*ChiP proteins. However, the OM-expressed protein and (to a lesser extent) in vitro-folded ChiP show short-lived channel closures that are absent in the N-terminally truncated channel (Fig. 7c), suggesting infrequent insertions of the plug inside the pore in the presence of a high and non-physiological membrane potential. To probe this further, we first induced closing of the OM-expressed *Vh*ChiP channels by applying a high voltage (+199 V; Fig. 7d). We then decreased the voltage to obtain very low membrane potentials (±2.5 mV; Fig. 7e). Under these conditions we observe long-lived channel closures that are fully consistent with the plug-inserted state observed in the crystal structure. Interestingly, three subconductance levels are observed corresponding to trimers with one, two or three open pores, indicating that the pore plugs move in and out of the channels independently. The channels open permanently when the voltage is increased to ±10 mV (Fig. 7f). The low voltages required for channel opening are somewhat surprising given the large free energy of plug binding derived from MD simulations (Fig. 3).

### A plug peptide blocks the pore of plug-less *Vh*ChiP

**Fig. 8:**
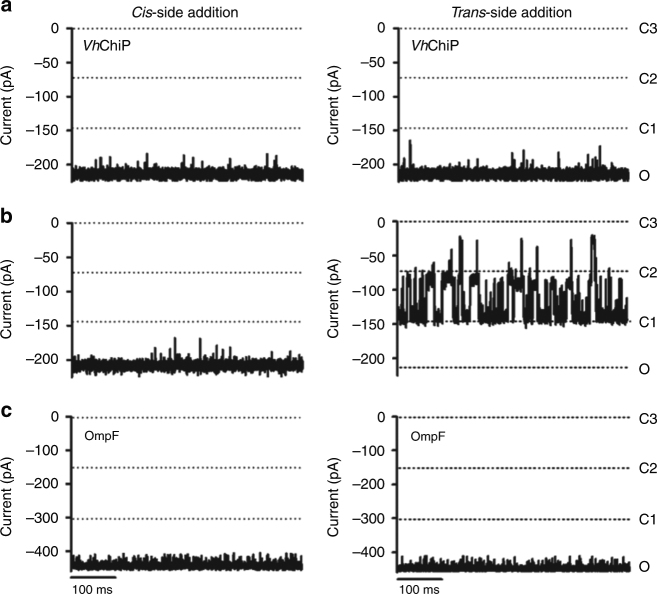
Channel blockage by an N-terminal plug peptide. Single-channel current traces at −100 mV (1 M KCl) of truncated VhChiP in the absence (**a**) and presence of 100 μM (**b**) of the plug peptide DGANSDAAK. **c** *E. coli* OmpF traces in the presence of 200 μM peptide. Traces shown are representative of three independent experiments

To probe the role of the pore plug further, we added a peptide corresponding to the first nine residues of *VhChiP* (DGANSDAAK) to the N-terminally truncated protein. At 100 μM peptide, frequent current blockages corresponding to the complete closure of a single channel are observed (Fig. 8). No blockages are observed for the control OmpF in the presence of 200 μM peptide. Interestingly, blockage of *Vh*ChiP is asymmetric, i.e., it occurs only upon peptide addition from one side (*trans*), regardless of the sign of the voltage (Supplementary Fig. 3). Given that proteins are added *cis* and preferentially insert into the lipid bilayer without translocating the long extracellular loops (i.e., these remain on the *cis* side), *trans* most likely corresponds to the periplasmic side of the channel. This experiment therefore recapitulates the physiological insertion of the pore plug. To further test specificity we also tested two unrelated peptides of similar length for *VhChiP* pore blockage. Neither of the control peptides affect the *Vh*ChiP-mediated currents (Supplementary Fig. 4), indicating that pore blockage is specific.

### Effect of the N-terminus on substrate binding and transport

**Fig. 9:**
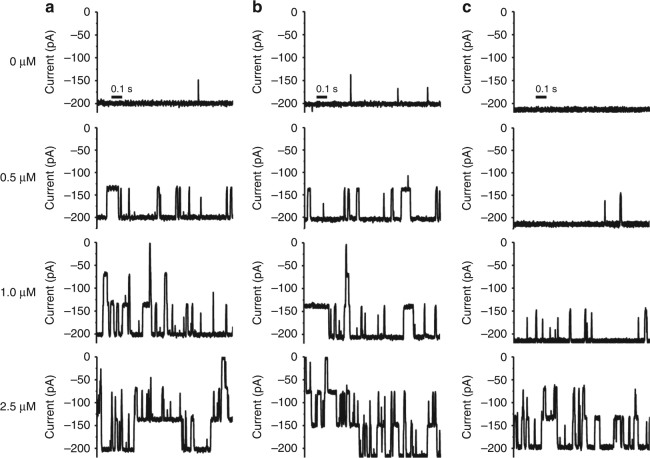
Chitoxexaose binds to *Vh*ChiP with high affinity. Single-channel electrophysiology experiments for natively expressed *Vh*ChiP (**a**), refolded *Vh*ChiP (**b**), and N-terminally truncated *Vh*ChiP (**c**). The fully open *Vh*ChiP trimeric channels were exposed to different concentrations of chitohexaose. Ion current traces were acquired at −100 mV with sugar added on the *cis* side. For full trace recordings of 2 min duration and analyses see Supplementary Figs. 3–6

Next, we tested the three proteins in the presence of increasing concentrations of chitohexaose (Fig. 9 and Supplementary Figs. 5–8). Under the conditions employed (−100 mV voltage) all three channels are permanently open (previous section). The results show that one subunit of the full-length channels was frequently blocked even at very low concentrations of chitohexaose (0.5 µM), while under the same conditions blocking events were rarely seen with the truncated channel. At 2.5 µM, occlusion of all three subunits was observed for the full-length channels, but not for truncated *Vh*ChiP. The data therefore suggest that the truncated channel interacts weaker with the sugar compared to the full-length channels. When the equilibrium binding constant *K* is evaluated, the full-length channels show relatively similar binding constants of ~2–5 × 10^5^ M^−1^ in agreement with previous results (Supplementary Table 2)^11,28^. The binding constants vary somewhat with different membrane potentials and are generally two- to threefold higher for the in vitro-folded protein for reasons that are not clear. However, removal of the first nine residues affects substrate binding significantly, with binding constants 10–15-fold lower for truncated *Vh*ChiP compared to OM-expressed, full-length protein (Supplementary Table 2).

**Fig. 10:**
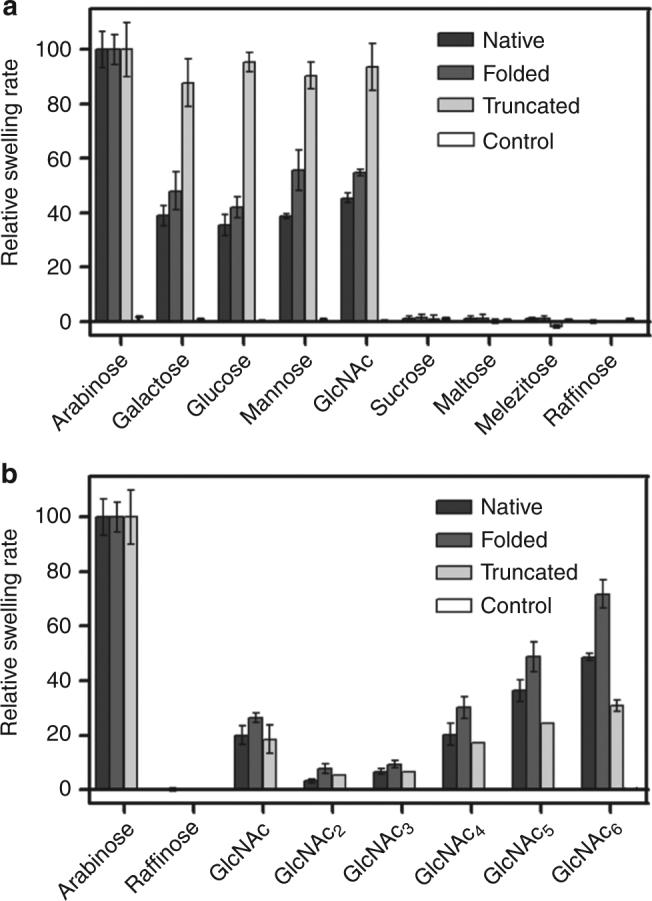
The N-terminus is important for chito-oligosaccharide substrate transport. Proteoliposome swelling assays in the presence of various mono- and oligosaccharides (**a**) as well as chito-oligosacharides of different lengths (**b**). No permeation of any sugars was observed when tested with control liposomes without *Vh*ChiP. “Folded” denotes in vitro-folded *Vh*ChiP. Data represent mean ± s.d., *n* = 3. For details see Methods

For most systems, a limitation of single-channel electrophysiology is the inability to distinguish substrate binding from substrate translocation through the channel, i.e., a compound can be released on the same side as where it binds. We therefore also assayed in vitro transport of various sugars via proteoliposome swelling assays. Various monomeric sugars translocate efficiently through *Vh*ChiP in the presence and absence of the N-terminus (Fig. 10). The truncated variant exhibited a more non-specific channel behavior and was approximately twofold more efficient in transport of various types of small monosaccharides compared to the full-length channels. By contrast, translocation of oligomeric sugars is exquisitely specific in accordance with previous results^10,11^, i.e., only chito-oligosaccharide substrates are translocated efficiently. Interestingly, for the oligosaccharides the absence of the N-terminus leads to a substantial decrease in transport rates, which is in qualitative agreement with the decreased binding constants observed in electrophysiology. Collectively the results suggest that the N-terminal segment plays an important role in chito-oligosaccharide substrate translocation through the *Vh*ChiP channel (Fig. 10).

## Discussion

The X-ray crystal structure of trimeric OM-purified *Vh*ChiP shows an unprecedented closing of the transport pore by the N-terminus of a neighboring monomer. Potential of mean force calculations suggest that this structural feature is highly stable in the absence of substrate. However, the applied-field MD simulations do not explain why the N-terminal plug is ejected at very low voltages in the experiments. These ejections might be rather slow processes involving details of the ion distributions and electro-osmotic forces on the plug that are currently out of scope for the present MD simulations due to the limited simulation time scales. In addition, the apparent discrepancy between the high computed binding free-energy of the plug and the measured low voltage of plug ejection might be due to differences in salt conditions, constant protonation states in the simulations and force-field approximations.

Interestingly, the single-channel experiments on truncated *Vh*ChiP in the presence of chitohexaose suggest that the absence of the N-terminus results in a weaker binding affinity of the substrate for the channel. Likewise, the proteoliposome swelling experiments show lower rates of chitooligosaccharide transport in the *Vh*ChiP variant lacking the N-terminus. These results have several implications. First, they would be difficult to reconcile with a scenario where the N-terminus would always be disordered and located outside the barrel, as observed in the crystal structure obtained from in vitro-folded *Vh*ChiP. Thus, the results suggest that N-terminal gating also occurs in the lipid bilayer, which is confirmed by single-channel experiments at very low membrane potential, approaching physiological conditions where the Donnan potential across the OM will be close to zero^36^. Second, the N-terminal gate is most likely not a passive structural feature but instead promotes substrate translocation. How this occurs is still unclear, but one possibility is that the N-terminal plug generates the optimal configuration of the substrate binding site. This hypothesis is given some support by the MD simulations, which show that the presence of the N-terminus results in increased dynamics of a region in loop L3 close to several substrate-interacting residues (Fig. 3a). This could be beneficial to substrate binding/orientation and consequently would make transport more efficient, providing support for the electrophysiology and in vitro transport data.

Is the putative gating mechanism conserved and why would there be a need for gating? Inspection of the sequence alignment (Fig. 5) shows that the N-terminal extension forming the pore plug is present only in a subset of *Vh*ChiP orthologs from *Vibrio* spp., indicating that other *Vh*ChiP orthologs may not be gated, or perhaps are gated in a different way. Regarding the need for a gating mechanism, in other enteric bacteria such as *E. coli* it is well established that the general porins OmpF and OmpC are not gated, but differently expressed depending on the osmolarity of the medium. At high osmolarity, expression of the smaller-diameter OmpC is upregulated relative to that of OmpF, presumably restricting the salt concentrations in the periplasmic space. *Vibrio* spp. also have orthologs of the general porins (named OmpU and OmpT in *V. cholerae*). While the structures of OmpU and OmpT have not yet been solved it is known that their expression is also regulated by osmolarity, with the larger-pore OmpU protein downregulated under conditions of high osmolarity^37^. Thus, a general picture emerges where under conditions of high osmolarity the entry of excess ions is restricted via upregulation of smaller-diameter channels and/or downregulation of larger ones. *Vh*ChiP is highly upregulated in marine environments in the presence of chitin^21^; for such a highly expressed channel, gating would be an effective means to restrict the entry of ions in a high-osmolarity environment. As mentioned above, how *Vh*ChiP orthologs with a shorter N-terminus (Fig. 5) would restrict ion access to the periplasmic space is not yet clear.

In conclusion, our studies on an important OM channel from marine *Vibrio* spp. highlight an emerging theme that substrate transport by OM channels is often more complicated than simple diffusion through a large, static pore. Ligand gating has been observed before in monomeric 14-stranded OM proteins^33,38^, but gating by a neighbor within an oligomeric assembly has, to our knowledge, not been reported in any membrane protein. Moreover, our results demonstrate that gating, rather than being a passive switch between open and closed states, might play a more active role in substrate transport. Future studies will be required to illuminate how gating improves substrate translocation and how ligands cause the necessary conformational changes that open the gate.

## Methods

### Cloning and expression of OM-expressed *Vh*ChiP

A BlastP search with chitoporin from *V. furnissii* as input (UniProtKB/TrEMBL entry: Q9KK91) identified VIBHAR_01269 as a putative chitoporin ortholog (GenBANK accession number YP_001444474) from the *V. harveyi* type strain ATCC BAA-1116 (also named *V. campbellii* ATCC BAA-1116). Therefore, oligonucleotides were designed for the gene from the BAA-1116 BB120 strain to clone the chitoporin gene from our laboratory strain (*V. harveyi* type strain 650). Genomic DNA was prepared using the PureLink Genomic DNA Kit (Invitrogen, Gibthai Company Ltd., Bangkok, Thailand) and used as the template for PCR. The oligonucleotides used for PCR are listed in Supplementary Table 3. The PCR product was of the expected size (1.1 kbp) and was cloned into pET23d(+) using *Nco I* and *Xho I* restriction sites, following the protocol of the manufacturer. Nucleotide sequences of both strands of the PCR product were determined by automated sequencing (First BASE Laboratories Sdn Bhd, Selangor Darul Ehsan, Malaysia). The recombinant plasmid harboring the *chiP* gene (pET23d(+)/*chiP*) was transformed into *E. coli* BL21(DE3) omp8 rosetta (Δ*lamB ompF*::Tn*5*Δ*ompA*Δ*ompC*^39^) (a gift from Prof. Roland Benz, Jacob University Bremen, Germany). The transformed cells were grown at 37 °C in Luria-Bertani (LB) liquid medium containing 100 μg mL^−1^ ampicillin, 25 μg mL^−1^ kanamycin and 1% (w/v) glucose. The expression was induced by adding 0.4 mM IPTG at an OD_600_ ~ 0.6 and the cells were incubated for 6 h at 37 °C. After cell disruption, the membranes were spun down and incubated with 2% (w/v) SDS for 1 h at 50 °C. Afterwards the membranes were washed with 0.125% (v/v) octyl-POE and finally extracted with 3% (v/v) octyl-POE. Insoluble particles were centrifuged for 30 min at 235,000 × *g* and the supernatant was loaded onto a HiPrep Mono Q anion exchange column. The column was washed with 10 column volumes (CV) 20 mM (Phosphate buffer) sodium phosphate and 0.2% (v/v) LDAO at pH 7.4. The protein was eluted with a linear gradient of 0–1 M KCl. Fractions containing *Vh*ChiP were pooled and subjected to size exclusion chromatography using a HiLoad 16/600 superdex 200 (GE Healthcare) using 10 mM HEPES, 100 mM LiCl, 0.4% (v/v) C_8_E_4_, pH 7.5. The purified protein was concentrated to 10–12 mg mL^−1^ and directly flash-frozen into liquid nitrogen prior to setting up crystallization trials.

The gene encoding for truncated *Vh*ChiP, lacking the first nineteen residues of the mature sequence (EVYGII--), was synthesized by GenScript and cloned into pET23a (Novagen). Subsequent protein expression and purification was carried out as described above for the full-length protein.

### Inclusion body expression of SeMet-labeled *Vh*ChiP

For expression of *Vh*ChiP into IBs, the gene without signal sequence and His-tag was cloned into pET28a (Novagen) via *Nco*I and *Xho*I (Supplementary Table 3). This procedure added the sequence “MG” to the N-terminus of the mature protein (MGDGANS--). The plasmid was transformed into BL21(DE3) cells (New England Biolabs). The cells were grown in minimal media (LeMasters-Richards) to OD_600_ ~ 0.6 at 37 °C before SeMet in combination with lysine, phenylalanine, threonine, leucine, isoleucine, and valine were added. Half an hour later the cells were induced with 1 mM IPTG at 37 °C for 3 h. Cells were harvested by centrifugation at 4500 × *g* for 30 min (Beckman Coulter). After cell disruption, IBs were harvested by centrifugation at 12,000 × *g* for 20 min and washed once in TBS with 1% Triton X-100 for 30 min at room temperature. This was followed by two washes without Triton X-100, followed by centrifugation for 20 min at 12,000 × *g* after each step. IBs were then solubilized at room temperature by stirring in ~25 mL TBS with 8 M Urea for 2 h. Non-solubilized pellet was removed by centrifugation at 200,000 × *g* for 30 min. For in vitro folding, the supernatant (~20 mL) was added dropwise to 200 mL TBS including 3% Elugent (Calbiochem) and the folding reaction was allowed to proceed at room temperature overnight with slow stirring. Subsequently, the folded protein was applied to a 10 mL anion exchange column and purified as described above for the OM-expressed protein.

### Crystallization and structure solution

Initial crystallization trials for OM-expressed *Vh*ChiP (10 mg mL^−1^) were performed at 295 K by sitting-drop vapor diffusion using MemGold1- and MemGold2-Screen from Molecular Dimensions with a mosquito robot (TTP Labtech). The initial hits were optimized by fine-screening with larger drops by hanging drop vapor diffusion. Crystals in space group C2 were grown in 28% (w/v) PEG 400, 0.2 M sodium acetate, 0.1 M MES pH 6.5. Crystals were directly flash-frozen in liquid nitrogen. A data set was collected at IO2 at the Diamond Light Source (DLS), UK. Se-Met crystals, derived from in vitro-folded *Vh*ChiP (~12 mg mL^−1^), were obtained in two different crystal forms. Crystal form I in space group P2_1_ was crystallized in 30% (w/v) PEG 400, 0.05 M NaCl, 0.1 M sodium citrate pH 5.5. Crystals in crystal form II were grown in 28% (w/v) PEG 400, 0.5 M potassium iodide, 0.1 M Tris pH 8.5. A SAD data set for crystal form I was collected to 1.95 Å at beamline IO2 at the Diamond Light Source (DLS), UK. Data were integrated and scaled with XDS^40^. Initial phasing and modeling was done using AUTOSOL within PHENIX^41^. Further model building was performed using the program COOT^42^. The protein model was refined with REFMAC^43^. Phases for OM-expressed *Vh*ChiP and in vitro-folded *Vh*ChiP in crystal form II were obtained by MR using MOLREP^44^ with the refined structure of SeMet-*Vh*ChiP as a search model. Model building was performed using COOT^42^ and the structure was refined with REFMAC^43^. The program MolProbity^45^ was used to evaluate the final model and PyMOL^46^ (Schrödinger, LLC) for the visualization of the protein structures.

For *Vh*ChiP complex structures, in vitro-folded protein (12 mg mL^−1^) was co-crystallized with 2.5 mM GlcNAc_6_ in 34% (w/v) PEG 400, 0.05 M NaCl, 0.1 M sodium citrate pH 5.5. Accordingly, OM-expressed protein (10 mg mL^−1^) was co-crystallized with 10 mM GlcNAc_4_ in  28% (w/v) PEG 400, 0.2 M sodium acetate, 0.1 M MES pH 6.5. The structures were solved via MR as described above for the apo proteins.

### MD simulations

The starting structures of the MD simulations were prepared as follows. The crystal structures of the *Vh*ChiP trimer in closed and open conformations were embedded in a 1-Palmitoyl-2-oleoyl-sn-glycero-3-phosphoethanolamine (POPE) bilayer together with TIP3P water and 1 M KCl salt. Subsequently, an energy minimization was performed using the steepest descent algorithm for 5000 steps followed by a 10 ns equilibration in the NVT and NPT ensembles with positional restraints on the heavy atoms of the proteins and lipids. The final unbiased simulations of *Vh*ChiP systems in open and closed conformations were performed for 500 ns. Furthermore, we carried out applied-field simulations for the in vitro-folded channel (200 mV: 3 × 250 ns each) and OM-expressed channel (200 mV: 3 × 250 ns each). For the free-energy calculations, we built an in silico* Vh*ChiP trimer variant with one mutation per monomer (D1A, D6A, and K9A) and a wild type system, both in the plug-inserted conformation, and including the necessary number of potassium ions to neutralize the system.

All MD simulations were performed with the GROMACS 4.6.5 program^47^ using the CHARMM36 all-atom force field^48,49^. The particle-mesh Ewald approach was used to calculate the long-range electrostatic interactions with a cutoff of 12 Å. Short-range Coulomb and Lennard Jones interactions were explicitly calculated up to the cutoff distance. The LINCS algorithm was applied to constraint the lengths of all bonds containing hydrogen atoms^50^. Moreover, the unbiased simulations discussed in this study were carried out in the NPT ensemble achieved by a semi-isotropic Parrinello-Rahman barostat^51^ at 1 bar with a coupling constant of 5 ps and the Nosé–Hoover thermostat^52,53^ with a coupling constant of 1 ps. The applied-field simulations were performed in a NVT ensemble with an electric field corresponding to transmembrane potential of 200 mV towards the periplasmic side of the channel. The field strength is proportional to the voltage *V*, i.e., *E* = *V*/*L*_*z*_, where* L*_*z*_ denotes the system length in *z* direction normal to the membrane. During the MD simulations snapshots were collected at every 10 ps and the MD results were analyzed using the tools available in GROMACS 4.6.5 package.

To estimate the energetics for unbinding of the N-terminus, two umbrella sampling calculations were performed for the wild type and the above-described trimer variant system. To save computational resources, we took advantage of the trimeric organization of the channel and simultaneously simulated the free energy profiles of the three mutant plugs (D1A, D6A, and K9A) along a single reaction coordinate. The one-dimensional reaction coordinate corresponded to the *z*-coordinate distance between the centers of mass (COM) of the N-terminal plug (Cα atoms of residues 1–10) and the corresponding barrel (Cα atoms of residues 25–350). The reaction coordinate was divided into 28 windows and we simulated each window for 90 ns with a harmonic potential of 1000 kJ mol^−1^ nm^−2^ on the reaction coordinate. The unbiased free energy profiles were calculated from the last 80 ns of each window trajectory using the g_wham^54^ implementation of the weighted histogram analysis method^55^.

### Single-channel electrophysiology

Planar lipid bilayer reconstitution was carried out in electrolyte containing 1 M KCl and 20 mM HEPES pH 7.5, at 25 °C^28,56^. Montal–Mueller type solvent-free bilayer formation was carried out using 1,2-diphytanoyl-sn-glycero-3-phosphatidylcholine (DPhPC; Avanti Polar Lipids, Alabaster, AL). First, the aperture was pre-painted with a few microliters of 1%(v/v) hexadecane in hexane, after which a planar bilayer was formed across the aperture by lowering and raising the liquid level. Ion currents were detected using Ag/AgCl electrodes with a patch-clamp amplifier connected to a two-electrode bilayer head-stage (PC-ONE plus PC-ONE-50; Dagan Corp., Minneapolis, MN, USA). The bilayer setup was operated within a Faraday cage on a vibration-dampened table, with an A/D converter (LIH 1600, HEKA Elektronik, Lambrecht, Germany) and was operated using the PULSE software program (HEKA Elektronik, Lambrecht, Germany). One of the electrodes, immersed in 1 M KCl electrolyte on the *cis* side of the cuvette, was connected to ground, while the electrode on the *trans* side was connected to the amplifier head-stage. *Vh*ChiP was always added to the *cis* side of the cuvette. Conductance values were determined from the *I*–*V* curves obtained from single-channel insertions at different voltages.

To investigate sugar translocation, single channels of the *Vh*ChiP variants were reconstituted in the artificial lipid membrane. To prevent multiple insertions during data acquisition, the protein solution in the chamber was gently diluted after the first insertion by sequential additions of working electrolyte. Subsequently the fully open channel was titrated with chitohexaose with concentrations ranging from 0.1 to 10 µM. Sugars were added to the *cis* side of the chamber. Ion flow was usually recorded for 2 min at different transmembrane potentials. The equilibrium binding constant *K* (M^−1^) was derived from the decrease in the ion conductance resulting from increasing concentrations of sugar using the following Equation (1) (ref. 57):

1 $$G_{{\mathrm{max}}} - G_{\mathrm{c}}/G_{{\mathrm{max}}} = I_{{\mathrm{max}}} - I_{\mathrm{c}}/I_{{\mathrm{max}}} = K[{c}]/(K[{c}] + 1),$$

*G*_max_ is the average conductance of the fully open *Vh*ChiP channel and *G*_c_ is the average conductance at a given concentration [*c*] of a chitooligosaccharide. *I*_max_ is the initial current through the fully open channel without sugar and *I*_c_ is the current at a particular sugar concentration.

### Liposome swelling experiments

*Vh*ChiP-reconstituted proteoliposomes were prepared using soybean l-α-phosphatidylchloline (20 mg mL^−1^, freshly prepared in chloroform) (Sigma-Aldrich) to form multi-lamellar liposomes^10^. Following this, 200 ng of *Vh*ChiP was reconstituted into 200 μL of liposome suspension by sonication, and 17% (w/v) dextran (40 kDa) was entrapped in the proteoliposomes by drying under vacuum and resuspending in 10 mM phosphate buffer pH 7.5. For determination of the isotonic solute concentration, d-Raffinose solutions were prepared in phosphate buffer with concentrations of 40, 50, 60, and 70 mM. The obtained isotonic concentration was then used for the adjustment of the isotonic concentration for other solutes. All small sugars (Fig. 10a) were prepared at 60 mM concentration. For chitooligosaccharides (Fig. 10b) we prepared the solutions differently, and used 1 mM of each chitosugar in 59 mM raffinose. The osmolarity of each sugar solution was checked to be at the isotonic concentration using a Genotec Osmometer 300. The reasons for using low concentrations of the chitosugars are twofold: (i) GlcNAc_4–6_ are not soluble at 60 mM and (ii) GlcNAc_4–6_ interact with *Vh*ChiP at low µM concentration (not mM). The liposome-swelling assays were carried out by adding 25 µL of proteoliposome suspension to 600 µL of sugar solution and the changes in absorbance at 500 nm were monitored immediately. The absorbance change over the first 60 s was used to estimate the swelling rate (s^−1^) according to: *φ* = (1/*A*_*i*_)d*A*/d*t*, in which *φ* is the swelling rate, *A*_*i*_ the initial absorbance, and d*A*/d*t* the rate of absorbance change during the first 60 s. The swelling rate for each sugar was normalized by setting the rate of l-arabinose (150 Da), the smallest sugar, to 100%. The values presented are averages from three to five independent experiments. The sugars tested were d-glucose (180 Da), d-mannose (180 Da), d-galactose (180 Da), *N*-acetylglucosamine (GlcNAc) (221 Da), d-sucrose (342 Da), d-melezitose (522 Da), GlcNAc_2_ (424 Da), GlcNAc_3_ (628 Da), GlcNAc_4_ (830 Da), GlcNAc_5_ (1034 Da), GlcNAc_6_ (1237 Da), and maltodextrins. Protein-free liposomes and proteoliposomes without sugars were used as negative controls.

### Data availability

The atomic coordinates and the associated structure factors have been deposited in the Protein Data Bank (http://www.pdbe.org) with accession codes 5MDO (In vitro-folded *Vh*ChiP, crystal form 1), 5MDP (In vitro-folded *Vh*ChiP, crystal form 2), 5MDQ (OM-expressed VhChiP), 5MDR (In vitro-folded *Vh*ChiP with chito-hexaose), and 5MDS (OM-expressed *Vh*ChiP with chito-tetraose). Other data supporting the findings of this study are available from the corresponding authors upon reasonable request.

## Electronic supplementary material

Supplementary Information

## References

[1] Klemm D (2005). Cellulose: fascinating biopolymer and sustainable raw material. Angew. Chem. Int. Ed..

[2] Muzzarelli, R. A. A. *Chitin* (Pergamon Press, UK, 1977).

[3] Gooday GW (1990). The ecology of chitin degradation. Adv. Microb. Ecol..

[4] Tracey MA (1957). Chitin. Rev. Pure Appl. Chem..

[5] Hirono I, Yamashita M, Aoki T (1998). Note: molecular cloning of chitinasegenes from *Vibrio anguillarum and V. parahaemolyticus*. J. Appl. Microbiol..

[6] Zobell CE, Rittenberg SC (1938). The occurrence and characteristics of chitinoclastic bacteria in the sea. J. Bacteriol..

[7] Hunt DE (2008). Conservation of the chitin utilization pathway in the *Vibrionaceae*. Appl. Environ. Microbiol..

[8] Jung BO, Roseman S, Park JK (2008). The central concept for chitin catabolic cascade in marine bacterium, *Vibrios*. Macromol. Res..

[9] Keyhani NO, Li XB, Roseman S (2000). Chitin catabolism in the marine bacterium *Vibrio furnissii*. Identification and molecular cloning of a chitoporin. J. Biol. Chem..

[10] Suginta W (2013). Molecular uptake of chitooligosaccharides through chitoporin from the marine bacterium *Vibrio harveyi*. PLoS ONE.

[11] Suginta W (2013). Chitoporin from *Vibrio harveyi*, a channel with exceptional sugar specificity. J. Biol. Chem..

[12] Keyhani NO, Roseman S (1996). The chitin catabolic cascade in the marine bacterium *Vibrio furnissii*. Molecular cloning, isolation, and characterization of a periplasmic chitodextrinase. J. Biol. Chem..

[13] Suginta W (2010). Novel *β*-N-acetylglucosaminidases from *Vibrio harveyi* 650: cloning, expression, enzymatic properties, and subsite identification. BMC Biochem..

[14] Keyhani NO, Roseman S (1996). The chitin catabolic cascade in the marine bacterium *Vibrio furnissii*. Molecular cloning, isolation, and characterization of a periplasmic beta-N-acetylglucosaminidase. J. Biol. Chem..

[15] Bassler BL (1991). Chitin utilization by marine bacteria. Chemotaxis to chitin oligosaccharides by *Vibrio furnissii*. J. Biol. Chem..

[16] Keyhani NO (1996). The chitin catabolic cascade in the marine bacterium *Vibrio furnissii*. Characterization of an N,N′-diacetyl-chitobiose transport system. J. Biol. Chem..

[17] Bouma CL, Roseman S (1996). Sugar transport by the marine chitinolytic bacterium *Vibrio furnissii*. Molecular cloning and analysis of the glucose and N-acetylglucosamine permeases. J. Biol. Chem..

[18] Park JK, Keyhani NO, Roseman S (2000). Chitin catabolism in the marine bacterium *Vibrio furnissii*. Identification, molecular cloning, and characterization of A N,N′-diacetylchitobiose phosphorylase. J. Biol. Chem..

[19] Li X, Roseman S (2004). The chitinolytic cascade in *Vibrios* is regulated by chitin oligosaccharides and a two-component chitin catabolic sensor/kinase. Proc. Natl Acad. Sci. USA.

[20] Suginta W (2004). An endochitinase A from *Vibrio carchariae*: cloning, expression, mass and sequence analyses, and chitin hydrolysis. Arch. Biochem. Biophys..

[21] Meibom KL (2004). The *Vibrio cholerae* chitin utilization program. Proc. Natl Acad. Sci. USA.

[22] Schmitt EK, Vrouenraets M, Steinem C (2006). Channel activity of OmpF monitored in nano-BLMs. Biophys. J..

[23] Benz R (1986). Pore formation by LamB of *Escherichia coli* in lipid bilayer membranes. J. Bacteriol..

[24] Schülein K, Benz R (1990). LamB (maltoporin) of *Salmonella typhimurium*: isolation, purification and comparison of sugar binding with LamB of *Escherichia coli*. Mol. Microbiol..

[25] Suginta W, Smith MF (2013). Single-molecule trapping dynamics of sugar-uptake channels in marine bacteria. Phys. Rev. Lett..

[26] Suginta W, Winterhalter M, Smith MF (2016). Correlated trapping of sugar molecules by the trimeric protein channel chitoporin. Biochim. Biophys. Acta-Biomembr..

[27] Zachariae U (2006). High resolution crystal structures and molecular dynamics studies reveal substrate binding in the porin Omp32. J. Biol. Chem..

[28] Chumjan W (2015). Chitoporin from the marine bacterium *Vibrio harveyi*: probing the essential roles of Trp136 at the surface of the constriction zone. J. Biol. Chem..

[29] Moraes TF (2007). An arginine ladder in OprP mediates phosphate-specific transfer across the outer membrane. Nat. Struct. Mol. Biol..

[30] Hayat S (2016). Inclusion of dyad-repeat pattern improves topology prediction of transmembrane β-barrel proteins. Bioinformatics.

[31] Tsirigos KD, Elofsson A, Bagos PG (2016). PRED-TMBB2: improved topology prediction and detection of beta-barrel outer membrane proteins. Bioinformatics.

[32] Holm L, Rosenstrom P (2010). Dali server: conservation mapping in 3D. Nucleic Acids Res..

[33] van den Berg B (2015). Outer-membrane translocation of bulky small molecules by passive diffusion. Proc. Natl Acad. Sci. USA.

[34] Dutzler R, Wang YF, Rizkallah P, Rosenbusch JP, Schirmer T (1996). Crystal structures of various maltooligosaccharides bound to maltoporin reveal a specific sugar translocation pathway. Structure.

[35] van Straaten KE (2007). Structure of *Escherichia coli* lytic transglycosylase MltA with bound chitohexaose: implications for peptidoglycan binding and cleavage. J. Biol. Chem..

[36] Sen K, Hellman J, Nikaido H (1988). Porin channels in intact cells of *Escherichia coli* are not affected by Donnan potentials across the outer membrane. J. Biol. Chem..

[37] Chakrabarti SR (1996). Porins of *Vibrio cholerae*: purification and characterization of OmpU. J. Bacteriol..

[38] Lepore BW (2011). Ligand-gated diffusion across the bacterial outer membrane. Proc. Natl Acad. Sci. USA.

[39] Prilipov A, Phale PS, Van Gelder P, Rosenbusch JP, Koebnik R (1998). Coupling site-directed mutagenesis with high-level expression: large scale production of mutant porins from *E. coli*. FEMS Microbiol. Lett..

[40] Kabsch W (2010). Integration, scaling, space-group assignment and post-refinement. Acta Crystallogr. D Biol. Crystallogr..

[41] Adams PD (2010). PHENIX: a comprehensive Python-based system for macromolecular structure solution. Acta Crystallogr. D Biol. Crystallogr..

[42] Emsley P, Cowtan K (2004). Coot: model-building tools for molecular graphics. Acta Crystallogr. D Biol. Crystallogr..

[43] Murshudov GN (2011). *REFMAC5* for the refinement of macromolecular crystal structures. Acta Crystallogr. D Biol. Crystallogr..

[44] Vagin A, Teplyakov A (1997). MOLREP: an automated program for molecular replacement. J. Appl. Crystallogr..

[45] Chen VB (2010). MolProbity: all-atom structure validation for macromolecular crystallography. Acta Crystallogr. D Biol. Crystallogr..

[46] The PyMOL Molecular Graphics System, v.1.8 (Schrödinger, LLC., 2017).

[47] Pronk S (2013). Gromacs 4.5: a high-throughput and highly parallel open source molecular simulation toolkit. Bioinformatics.

[48] Klauda JB (2010). Update of the CHARMM all-atom additive force field for lipids: validation on six lipid types. J. Phys. Chem. B.

[49] Best RB (2012). Optimization of the additive CHARMM All-atom protein force field targeting improved sampling of the backbone φ, ψ and Side-chain χ1 and χ2 dihedral angles. J. Chem. Theory Comput..

[50] Hess B, Bekker H, Berendsen HJC, Johannes GEMF (1997). LINCS: a linear constraint solver for molecular simulations. J. Comput. Chem..

[51] Parrinello M, Rahman A (1981). Polymorphic transitions in single crystals: a new molecular dynamics method. J. Appl. Phys..

[52] Nosé SA (1984). Molecular dynamics method for simulations in the canonical ensemble. Mol. Phys..

[53] Hoover WG (1985). Canonical dynamics: equilibrium phase-space distributions. Phys. Rev. A.

[54] Hub JochenS, de Groot BL, van der Spoel D (2010). g_whams: a free weighted histogram analysis implementation including robust error and autocorrelation estimates. J. Chem. Theory Comput..

[55] Kumar S, Bouzida D, Swendsen RH, Kollman PA, Rosenberg JM (1992). The weighted histogram analysis method for free-energy calculations on biomolecules. I. The method. J. Comput. Chem..

[56] Schulte A (2009). The outer membrane protein VhOmp of *Vibrio harveyi*: pore-forming properties in black lipid membranes. J. Membr. Biol..

[57] Benz R, Hancock RE (1987). Mechanism of ion transport through the anion-selective channel of the *Pseudomonas aeruginosa* outer membrane. J. Gen. Physiol..

