# Supplementary material for: Structural basis for chitin acquisition by marine Vibrio species
Source: Nat Commun. 2018 Jan 15;9:220. doi: 10.1038/s41467-017-02523-y (PMC5768706; doi:10.1038/s41467-017-02523-y)
Supplement: Supplementary file 1 — Supplementary Information [file 41467_2017_2523_MOESM1_ESM.pdf]

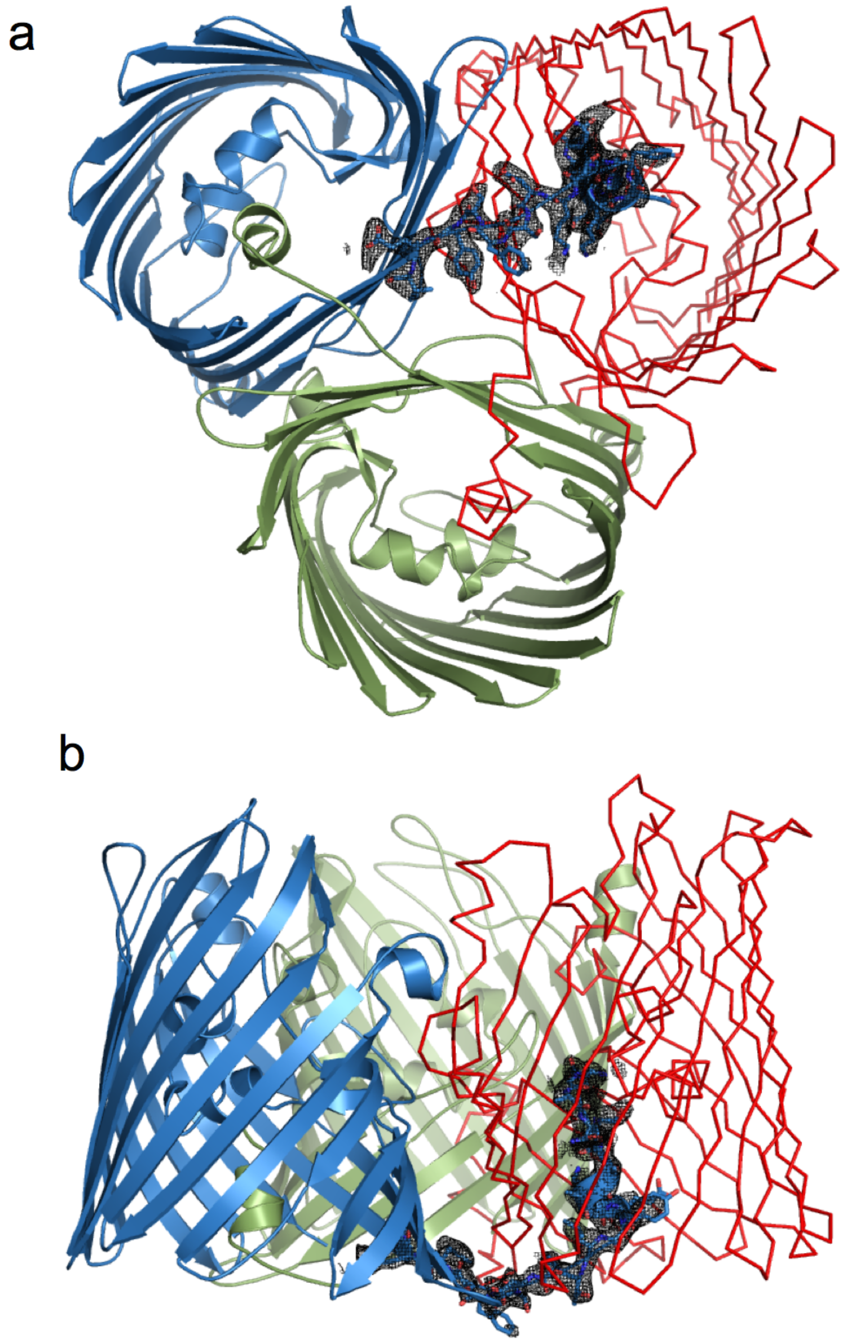

Supplementary Figure 1 Electron density of the *Vh*ChiP N-terminus. Unbiased Fo-Fc simulated annealing (SA) omit electron density map contoured at 3.0 σ (carve = 2.5 Å) viewed from the periplasmic space (**a**) and from the membrane plane (**b**), showing how the N-terminus of a monomer inserts into the channel of the neighbour. The map was calculated after refinement in Phenix with omission of the first 21 residues. Five cycles were performed, each with XYZ coordinates, TLS parameters, Occupancies and Individual B-factor refinement. In addition, cycles 2 and 4 included cartesian and torsion angle SA with a starting temperature of 1000 K. The electron density map (dark mesh) is shown for the omitted residues.

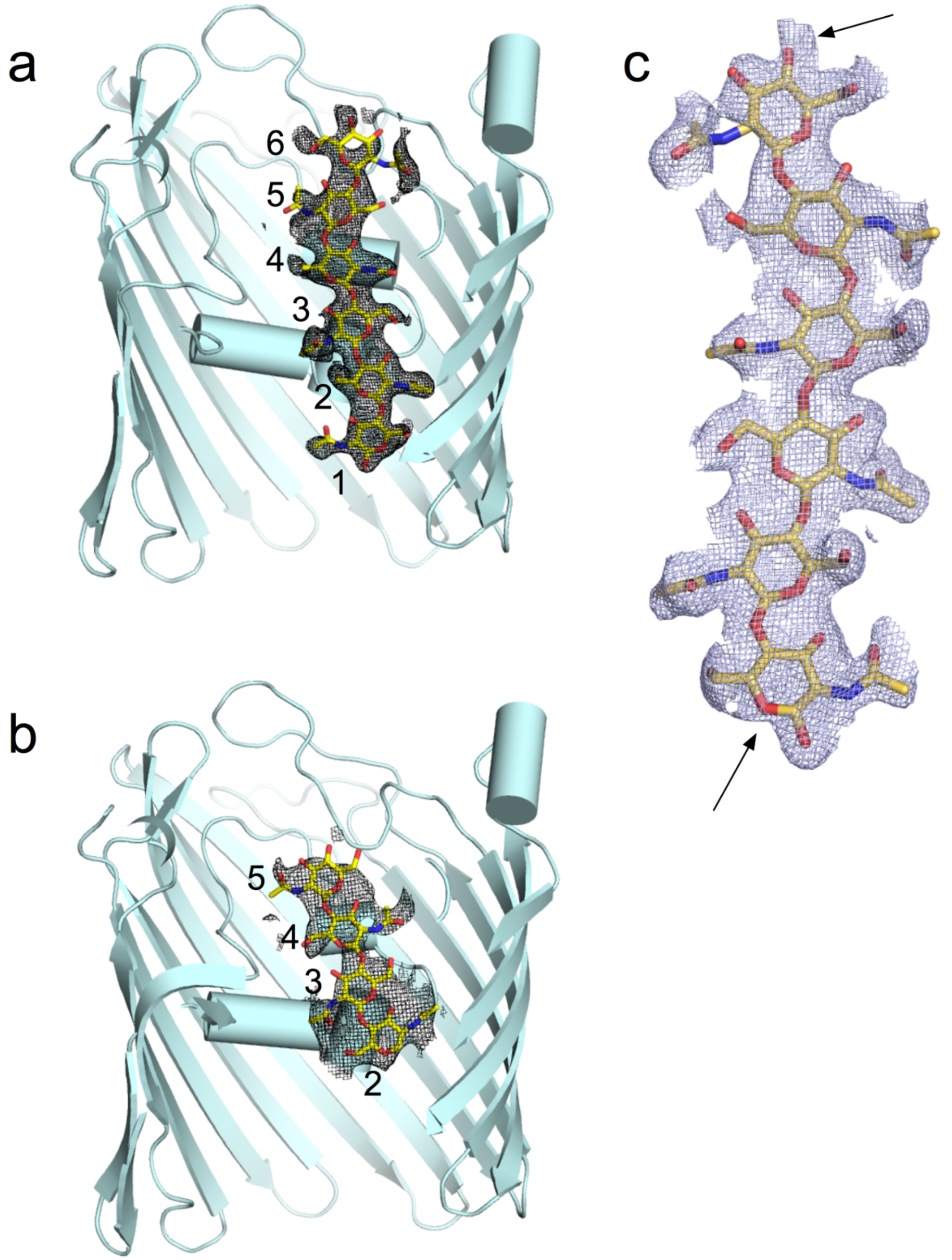

Supplementary Figure 2 Electron density of the chito-oligosaccharide substrates. Unbiased Fo-Fc SA omit electron density maps contoured at 3.0 σ (carve = 2.5 Å), for chitohexaose and *in vitro* folded *Vh*ChiP (**a**) and chitotetraose and OM-expressed *Vh*ChiP (**b**). Maps were calculated after 5 cycles of Phenix refinement with SA as described in Supplementary Fig. 1, with omission of the substrates. The sugar residues have been numbered. **c**, 2Fo-Fc density map of chitohexaose contoured at 0.8 σ (carve = 2 Å) supporting the assignment of the reducing end of the molecule (periplasmic side, bottom). This map was calculated in the presence of the substrate.

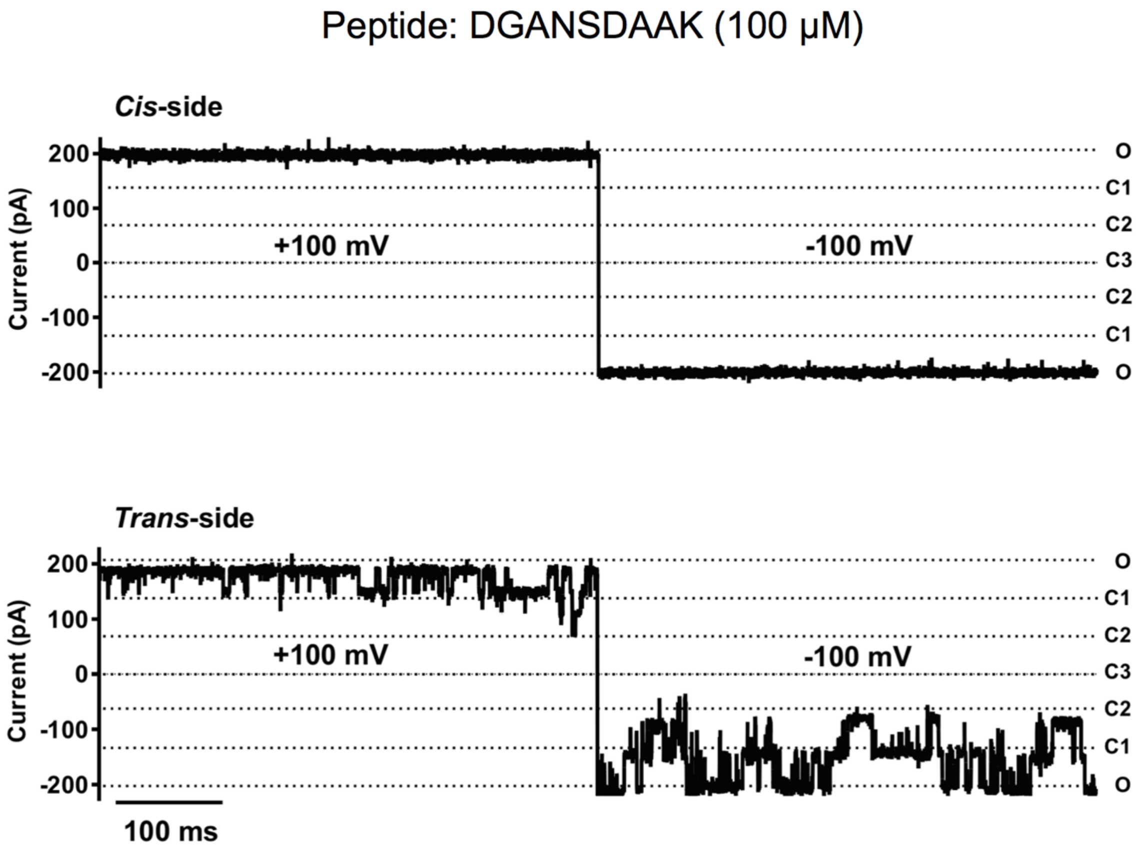

Supplementary Figure 3 Pore plug peptide blocks the *Vh*ChiP pore irrespective of the sign of the voltage. Single channel currents for truncated *Vh*ChiP in the presence of 100 μM of the DGANSDAAK peptide at +/- 100 mV. Peptide was added *cis* (top panels) or *trans* (bottom panels). Traces are representative of three experiments.

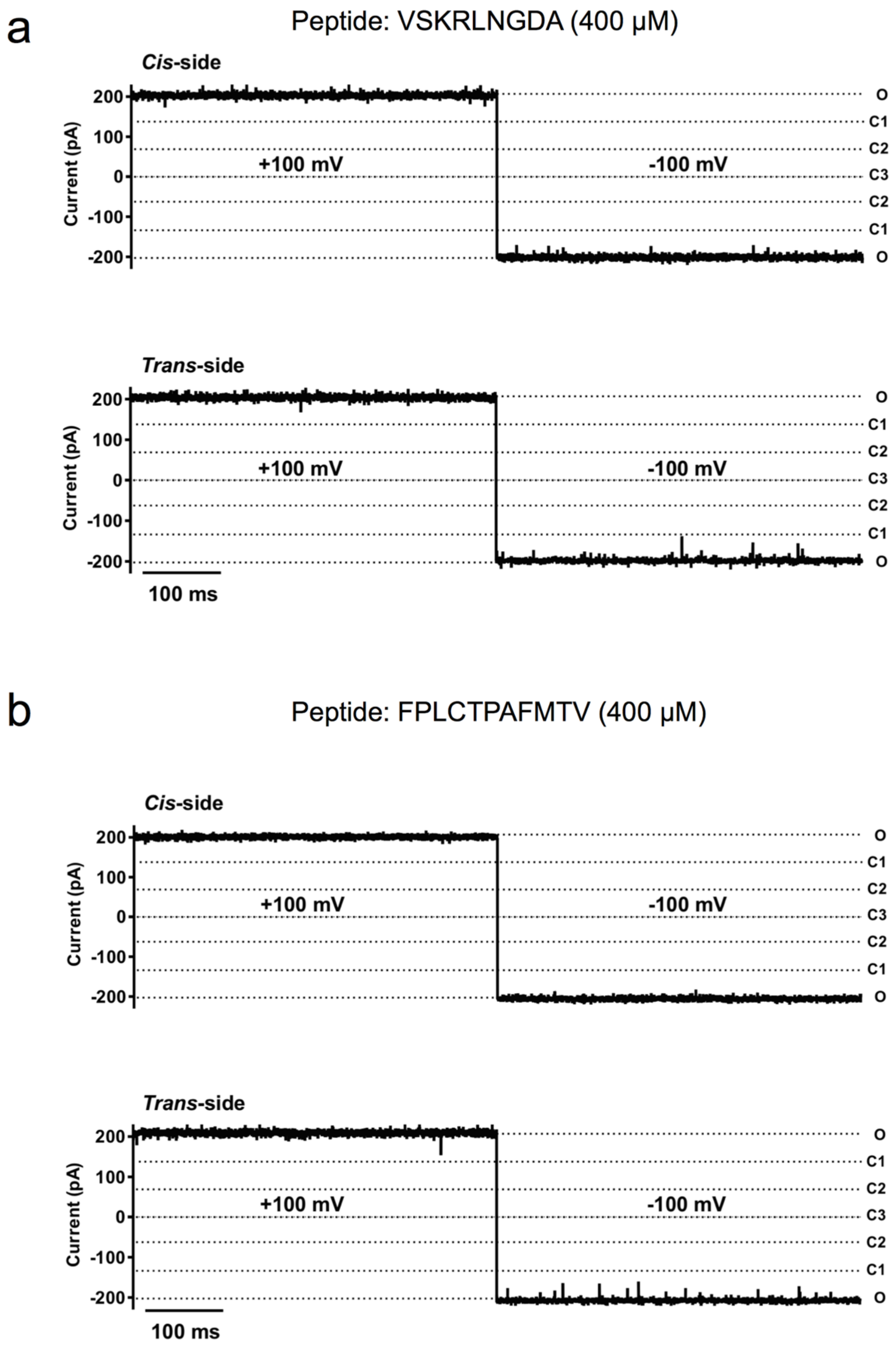

Supplementary Figure 4 Control peptides do not block the *Vh*ChiP pore. Single channel currents for truncated *Vh*ChiP in the presence of 400 μM of the peptides VSKRLNGDA (**a**) or FPLCTPAFMTV (**b**), at +/- 100 mV. Peptides were added *cis* (top panels) or *trans* (bottom panels). Traces are representative of three experiments.

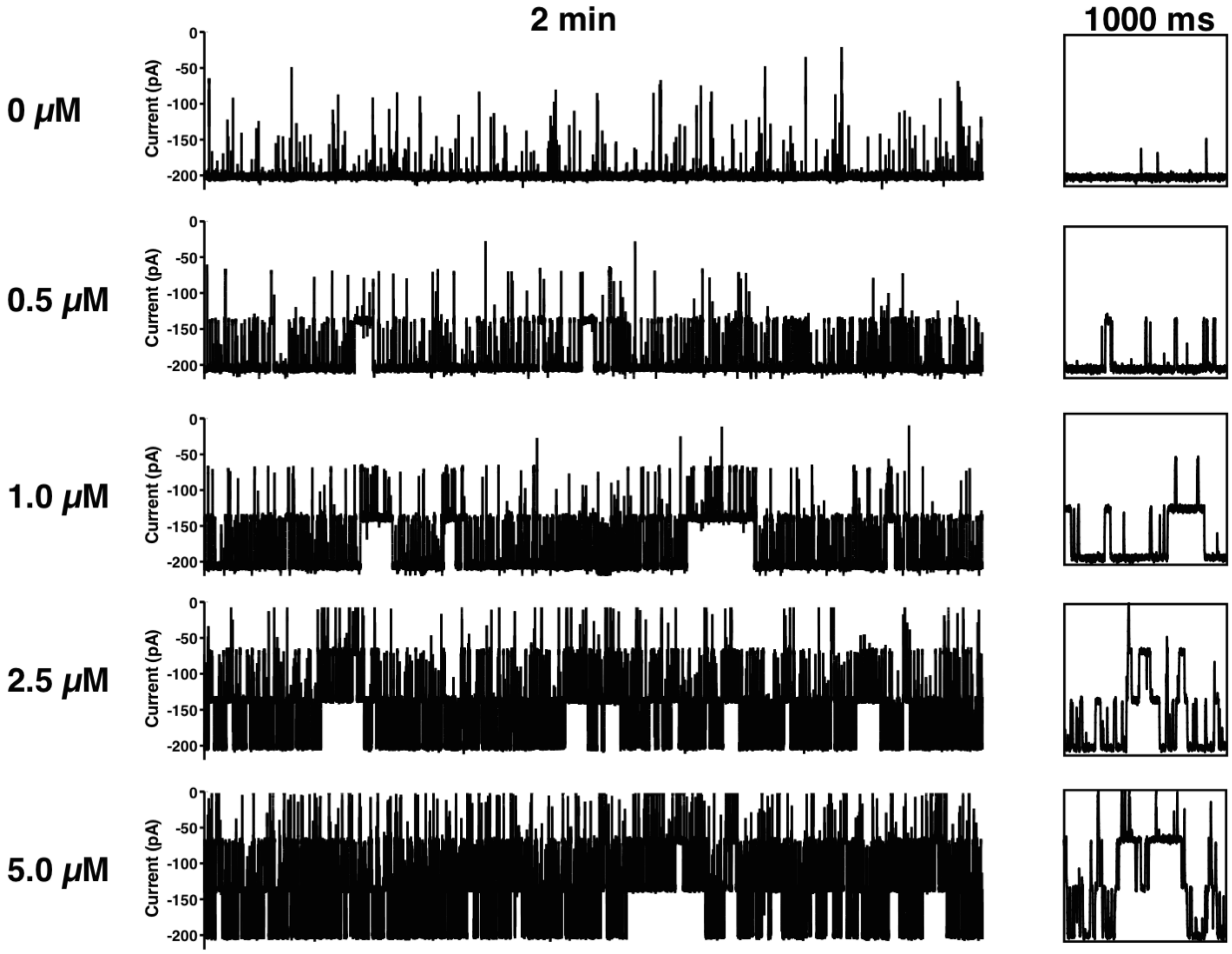

Supplementary Figure 5 Full time ion current recordings of native, OM-expressed *Vh*ChiP channels titrated with 0-5 μM chitohexose in 1 M KCl electrolyte. The channels were reconstituted into artificial phospholipid (DPhPC) bilayers as described in the text. After sugar was added on the cis side, the disturbance of ion flow by sugar blockade was recorded for 2 min at each applied voltage. The equilibrium binding constant (*K*) derived from the entire trace was estimated using noise analysis (ref. 57). The K values obtained from different voltages are presented in Table 1. For the sake of clarity, only the **I**(t) traces acquired at -100 mV are presented in this figure.

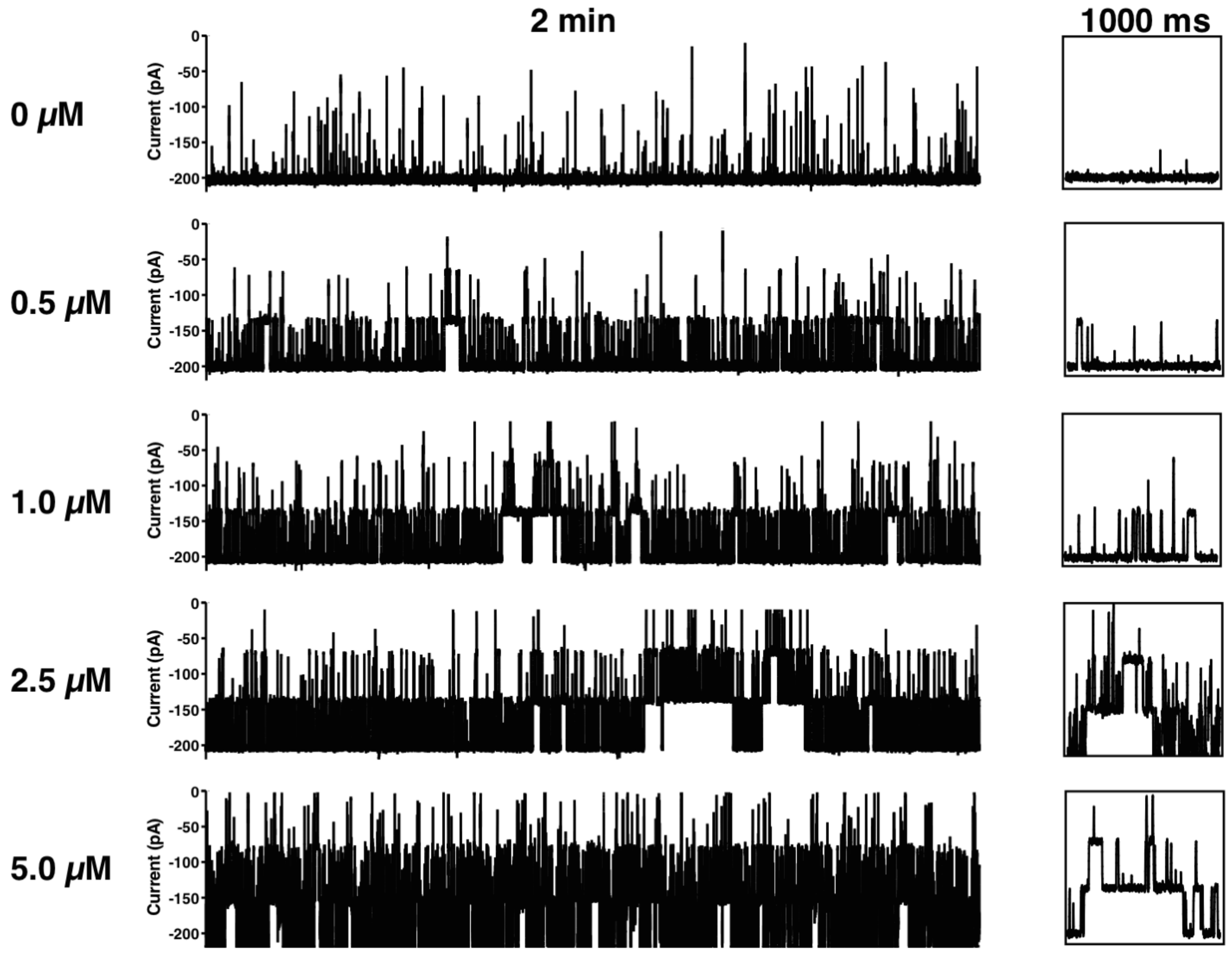

Supplementary Figure 6 Full time ion current recordings of *in vitro* folded *Vh*ChiP channels titrated with 0-5 μM chitohexose in 1 M KCl electrolyte. The channels were reconstituted into artificial phospholipid (DPhPC) bilayers as described in the text. After sugar was added on the cis side, the disturbance of ion flow by sugar blockade was recorded for 2 min at each applied voltage. The equilibrium binding constant (*K*) derived from the entire trace was estimated using noise analysis (ref. 57). The K values obtained from different voltages are presented in Table 1. For the sake of clarity, only the I(t) traces acquired at -100 mV are presented in this figure.

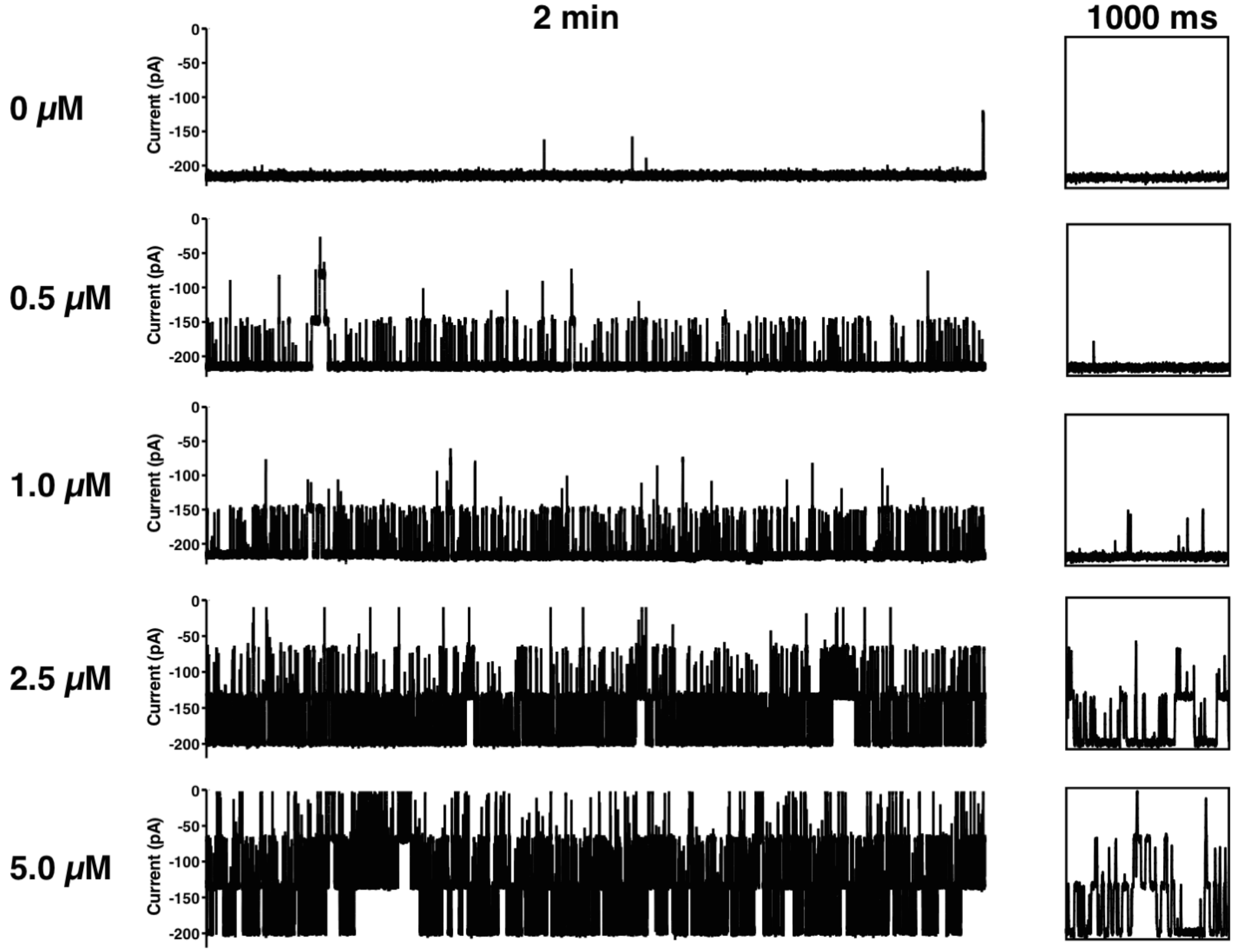

Supplementary Figure 7 Full time ion current recordings of truncated *Vh*ChiP channels titrated with 0-5 μM chitohexose in 1 M KCl electrolyte. The channels were reconstituted into artificial phospholipid (DPhPC) bilayers as described in the text. After sugar was added on the cis side, the disturbance of ion flow by sugar blockade was recorded for 2 min at each applied voltage. The equilibrium binding constant (*K*) derived from the entire trace was estimated using noise analysis (ref. 57). The K values obtained from different voltages are presented in Table 1. For the sake of clarity, only the I(t) traces acquired at -100 mV are presented in this figure.

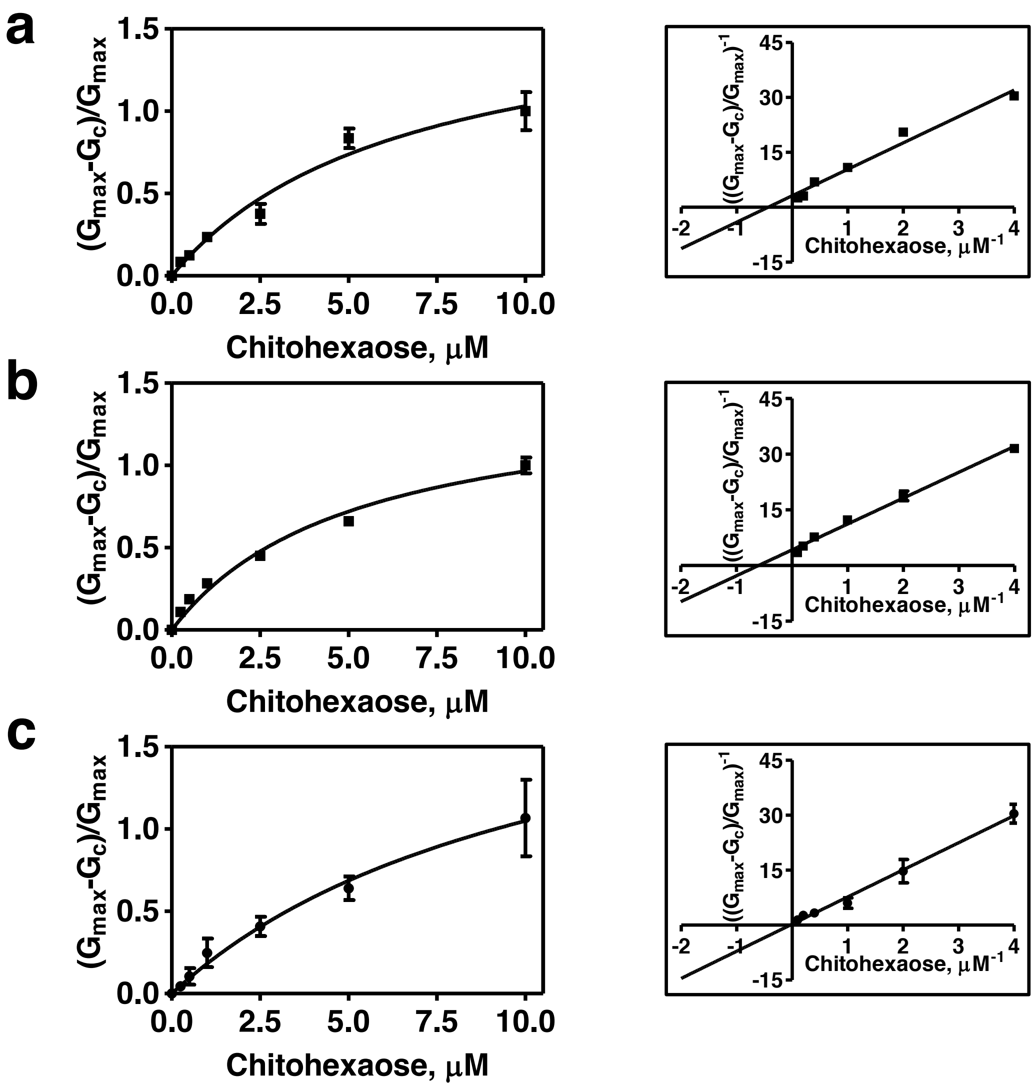

Supplementary Figure 8 Non-linear plots (right panel) derived from full trace recordings from OM-expressed (**a**), *in vitro* folded (**b**) and truncated *Vh*ChiP (**c**), as shown in Supplementary Figures 4-7. Left panels show linear curve fits of the non-linear plots used to determine the K values. Data represent mean ± s.d., n = 3. Both plots were generated by Prism v5.0.

Supplementary Table 1 Data Collection and Refinement statistics.

	In vitro folded *Vh*ChiP Crystal form I	*In vitro* folded *Vh*ChiP Crystal form II	OM-expressed *Vh*ChiP	*In vitro* folded *Vh*ChiP + chitohexaose	OM-expressed *Vh*ChiP + chitotetraose
**Data Collection**	DLS IO2	DLS IO4	DLS IO4-1	DLS IO2	DLS IO4
**Space Group**	$P2_1$	C2	C2	$P2_1$	C2
**Cell Dimensions**					
***a, b, c* (Å)**	146.9,123.4,147.0	254.8, 147.0, 53.8	255.7, 148.6, 54.4	146.6, 121.9, 147.2	250.0, 145.1, 55.2
***α, β, γ* (°)**	90, 117.9, 90	90, 95.5, 90	90, 94.9, 90	90, 117.9, 90	90, 94.8, 90
**Wavelength**	0.97949	0.97949	0.92	1.00727	0.97949
**Resolution (Å)**	64.97-1.95 (1.98-1.95)	29.21-3.08 (3.23-3.08)	49.15-2.5 (2.56-2.5)	64.75-1.90 (1.93-1.90)	62.68-2.60 (2.68-2.60)
**R_{merge} [%]**	7.2 (85.4)	10.8 (41.0)	8.6 (63.6)	6.2 (68.2)	9.0 (77.7)
**R_{pim} [%]**	4.6 (54.3)	10.0 (38.5)	7.8 (58.0)	5.6 (59.8)	8.1 (69.9)
**$\langle I/\sigma I \rangle$**	13.9 (1.9)	6.6 (1.9)	12.2 (2.1)	11.0 (1.8)	8.4 (1.1)
CC1/2	0.998 (0.777)	0.988 (0.804)	0.997 (0.729)	0.997 (0.670)	0.995 (0.530)
**Completeness (%)**	100.0 (100.0)	95.7 (92.8)	95.5 (97.5)	99.6 (99.5)	93.4 (95.1)
**Redundancy**	6.8 (6.7)	2.7 (2.7)	4.1 (4.1)	3.8 (3.7)	2.9 (2.9)
**Refi*nem*ent**					
**R_{work} / R_{free}**	16.7 / 18.7	21.7 / 24.0	23.0 / 24.1	17.2 / 18.4	21.9 / 24.5
**Ramachandran plot**					
**most favored [%]**	92.2	85.3	90.1	92.1	91.4
**No. atoms**					
**Protein**	16038	7995	8169	16002	7995
**Water**	1796	-	-	1748	-
**C8E4**	241	-	-	201	-
***B*-factors**					
**Protein**	37.2	71.2	56.2	33.1	62.3
**Water**	47.3	-	-	43.9	-
**C8E4**	59.2	-	-	55.1	-
**R.m.s. deviations**					
**Bond lengths (Å)**	0.02	0.02	0.02	0.01	0.02
**Bond angles (°)**	1.86	2.17	1.92	1.62	2.40
**pdb-code**	5MDO	5MDP	5MDQ	5MDR	5MDS

Supplementary Table 2 Equilibrium binding constants for chitohexaose and *Vh*ChiP variants.

**Voltage (mV)**	**Equilibrium binding constant (*K*, M^{-1})**		
	**Native *Vh*ChiP**	**Refolded *Vh*ChiP**	**Truncated *Vh*ChiP**
**+100**	245,000 ±35,000	424,000 ±65,000	34,000 ±7,200
**-100**	434,000 ±20,000	602,000 ±53,000	97,000 ±31,000
**+50**	154,000 ±52,000	309,000 ±64,000	19,400 ±8,500
**-50**	386,000 ±66,000	591,000 ±11,000	35,000 ±24,000
**+25**	103,000 ±58,000	287,000 ±38,000	7,400 ±2,800

Binding constants *K* were obtained by titrating 0- 5 μM of chitohexaose to single channels of the three *Vh*ChiP variants reconstituted into DPhPc bilayers on the HeKA BLM setup (See Methods). The values are mean ± SD, obtained from at least three independent measurements.

Supplementary Table 3 Primer sequences for cloning *Vh*ChiP

_Vh_ChiP OM expression

Forward Primer (*NcoI*)

5’ ATACCATGGCGTCTTACCTAAAGAAAAG 3’

Reverse Primer (*XhoI*)

5’-AACCTCGAGTTAGAAGTAGTATTCAACAC-3’

_Vh_ChiP Inclusion body expression

Forward Primer (*NcoI*)

5’ GCGCCATGGGCGATGGTGCAAACAGTG 3’

Reverse Primer (*XhoI*)

5’ GCGCTCGAGTTAGAAGTAGTATTCAAC 3’
